# Multiple attention based deep multimodal fusion network for glaucoma and neurodegenerative disease diagnosis

**DOI:** 10.1038/s41598-026-46855-6

**Published:** 2026-04-26

**Authors:** Md Mahmudul Hasan, Jack Phu, Henrietta Wang, Michael Kalloniatis, Arcot Sowmya, Erik Meijering

**Affiliations:** 1 School of Computer Science and Engineering, University of New South Wales, Sydney, NSW 2052 Australia; 2 School of Optometry and Vision Science, University of New South Wales, Sydney, NSW Australia; 3 Centre for Eye Health, University of New South Wales, Sydney, NSW Australia; 4 Faculty of Medicine and Health, University of Sydney, Camperdown, NSW Australia; 5 School of Medicine (Optometry), Deakin University, Waurn Ponds, VIC Australia; 6 University of Houston College of Optometry, University of Houston, Houston, TX USA

**Keywords:** 24–2 test grid, Colour fundus photographs, Glaucoma, Dementia, Parkinson’s disease, Perimetry, Visual fields, Optic nerve diseases, Biomedical engineering

## Abstract

Glaucoma and other neurodegenerative diseases involve progressive damage to the optic disc (OD) and can be identified using visual field (VF) tests, colour fundus photographs (CFP) and optical coherence tomography (OCT). However, most studies overlook neurodegenerative cohorts and primarily rely on unimodal approaches, thus failing to leverage advanced deep multimodal fusion techniques to capture modality-specific features and cross-modal interactions. To address the issue, this study aims to design a multi-attention-based deep multimodal network to fuse functional VF with structural CFP and OCT. Using data from 354 eyes with neuropathies (glaucoma, ischaemic optic neuropathy, dementia and Parkinson’s) and 228 normal eyes, we developed novel deep learning techniques for disc segmentation, fovea localisation, disc-fovea angle calculation and alignment, and multi-attention-based deep intermediate multimodal fusion incorporating different combinations of spatial, self- and cross-modal attention mechanisms. The results indicate that our proposed multi-attention-multimodal fusion network (MAM-Fusion-Net) achieved an area under the sensitivity–specificity curve (AUC) of 0.998 (95% CI 0.996–1.000) for diagnosing neuropathy versus normal participants and 0.981 (95% CI 0.966–0.996) for glaucoma versus non-glaucoma, surpassing state-of-the-art uni- and multi-modal models. This multi-attention-based deep fusion strategy applied to multimodal images shows potential in glaucoma and neurodegenerative disease diagnosis to assist eye care practitioners.

**Supplementary Information:**

The online version contains supplementary material available at 10.1038/s41598-026-46855-6.

## Introduction

Glaucoma, a leading cause of blindness worldwide^1^, manifests as progressive and irreversible vision loss, significantly impacting patients’ quality of life^2^. It shares common features with other neurodegenerative conditions like dementia and Parkinson’s disease^3^, with evidence suggesting that glaucoma patients have a higher risk of developing neurodegenerative diseases, due to similarities in neuronal loss and degenerative changes in ganglion cells^4^. According to Yu et al.^5^, 23% of Parkinson’s patients have a higher risk of getting affected by glaucoma, compared to the age-matched normal (5–12%). Also, the study has reported that dementia patients have a higher incidence of developing glaucoma in late life^5^.

Glaucoma is characterised by gradual damage to the visual field (VF)^6,7^, leading to structural changes such as retinal nerve fiber layer (RNFL) loss^8,9^. Recent studies suggest characteristic changes in VF for neurodegenerative patients. Studies report that dementia patients due to Alzheimer’s have significantly higher VF loss than other patients with dementia^10,11^, the existence of a bilateral ‘glaucoma-like’ VF defect in Parkinson’s disease compared to the control cohort^12,13^, and VF defects in mostly nasal region for ischaemic optic neuropathy (ION) patients^14^.

A key method for the diagnosis of glaucoma is colour fundus photography (CFP), a cost-effective and accessible method for capturing optic disc (OD) characteristics^15,16^. Being correlated with ganglion cell loss, neurodegenerative patients such as dementia and Parkinson’s also show certain characteristic changes in CFP, such as increased vessel width variability^17^ attenuated branching complexity and geometrical pattern^17^, increased tortuosity^18,19^ and reduced vessel calibre^18,20^.

Both fundoscopy, including CFPs, and VFs have unique strengths and limitations in glaucoma assessment, capturing different aspects of the disease^21^. Considering this, clinicians use both modalities concurrently for glaucoma diagnosis and management. However, given the large glaucoma population, it is a challenging task to do it manually, which is also time-consuming and expensive and may be prone to human error or higher variability between observations^22^. In addition to this, the presence of other optic neuropathies, such as ION and neurodegenerative diseases such as dementia, makes glaucoma challenging to detect, which causes half of the total population diagnosed with glaucoma to be undiagnosed^23^ and 60% of the population being over-diagnosed^24^. To address this issue, artificial intelligence (AI) methods applied to these modalities have recently shown promise in aiding clinicians in diagnosis. However, a recent review of the AI-based studies on glaucoma diagnosis found that most of these use unimodal data, causing less robust and reliable models, and hindering real-life applicability, which creates a potential scope for multimodal analysis^3^.

Only a handful of studies worked on multimodal data for glaucoma diagnosis^25^. However, these studies did not consider several aspects, such as the presence of neurodegenerative diseases, optic disc cropping, disc-fovea angle, and OD and VF alignment. First, regarding the OD cropping, an analysis of 492,023 fundus images by Gonzalez-Hernandez et al.^26^ revealed that the median optic disc area is 1.95 mm^2^, with the 1st and 99th percentiles ranging from 1.29 mm^2^ to 3.03 mm^2^. Using routine CFP, the optic disc covers only a limited portion of the overall fundus photograph, even at its largest^26^. However, higher magnification settings can be used to focus more specifically on the OD, providing greater detail, though this approach is not without limitations. Feeding a whole CFP to a deep learning (DL) network increases the model’s complexity, but also irrelevant information in the periphery of the image, which can introduce noise and potentially confuse the model, leading to misdiagnosis^27^.

Second, for multimodal fusion involving VF and CFPs, disc-fovea angle identification and alignment are crucial for multimodal studies utilising these two modalities. Notably, Abe et al.^28^ demonstrated that the disc-fovea angle is associated with the location of visual field defects in glaucoma patients, with less negative angles observed in eyes with central visual field defects compared to peripheral defects. Matos et al.^29^ conducted a cross-sectional study to examine how the foveal position relative to the optic disc affects 10–2 visual field (VF) outcomes in glaucoma patients with localised inferotemporal (IT) neuroretinal rim defects. Their findings revealed that vertical foveal position, along with its alignment relative to the IT neuroretinal rim defects, has a significant impact on the severity of VF defects, especially in certain sectors of the 10–2 grid. These findings underscore the importance of disc-fovea angle alignment in accurately assessing central visual fields, particularly in the early stages of glaucoma; however, this is not practised in the existing AI-based studies. To address the limitation, this study uses a novel approach for disc-fovea alignment before multimodal fusion of CFPs with VF images.

Third, a major issue in deep multimodal fusion is that previous multimodal studies predominantly focused on early or late fusion, which cannot capture modality-specific features and cross-modal interactions respectively^30–32^. These limitations of conventional early and late fusion approaches have motivated the development of more advanced fusion techniques that aim to balance modality-specific processing and cross-modal integration^2,4^. In our study, we address this gap by implementing an intermediate fusion strategy, supported by attention mechanisms^33^. We employed uni-, dual- and triple-attention mechanisms, and their combinations applied to the data, and developed a novel efficient and optimised architecture named multi-attention-based multimodal fusion network (MAM-Fusion-Net). Fourth, while optical coherence tomography (OCT)-based unimodal studies achieved reasonable performance for glaucoma diagnosis using extracted features such as RNFL thickness^34–37^, a fusion of OCT with CFP and VFs could improve the performance of the diagnosis. Considering this, we performed a sub-analysis fusing OCT with VF and CFP images.

To the best of our knowledge, this is the first study to use a comparative approach with multi-attention-based multimodal fusion applied to neuropathy diagnosis using CFP, VFs and OCT data, especially with a patient cohort including glaucoma and neurodegenerative diseases, such as dementia and Parkinson’s disease and ION. This approach offers a balance between the flexibility of modality-specific feature extraction and the ability to capture complex cross-modal dependencies, making it particularly well-suited for multimodal tasks, especially using structural (e.g. CFPs and OCT) and functional (e.g. VF) data for neuropathy detection.

## Materials and methods

**Fig. 1:**
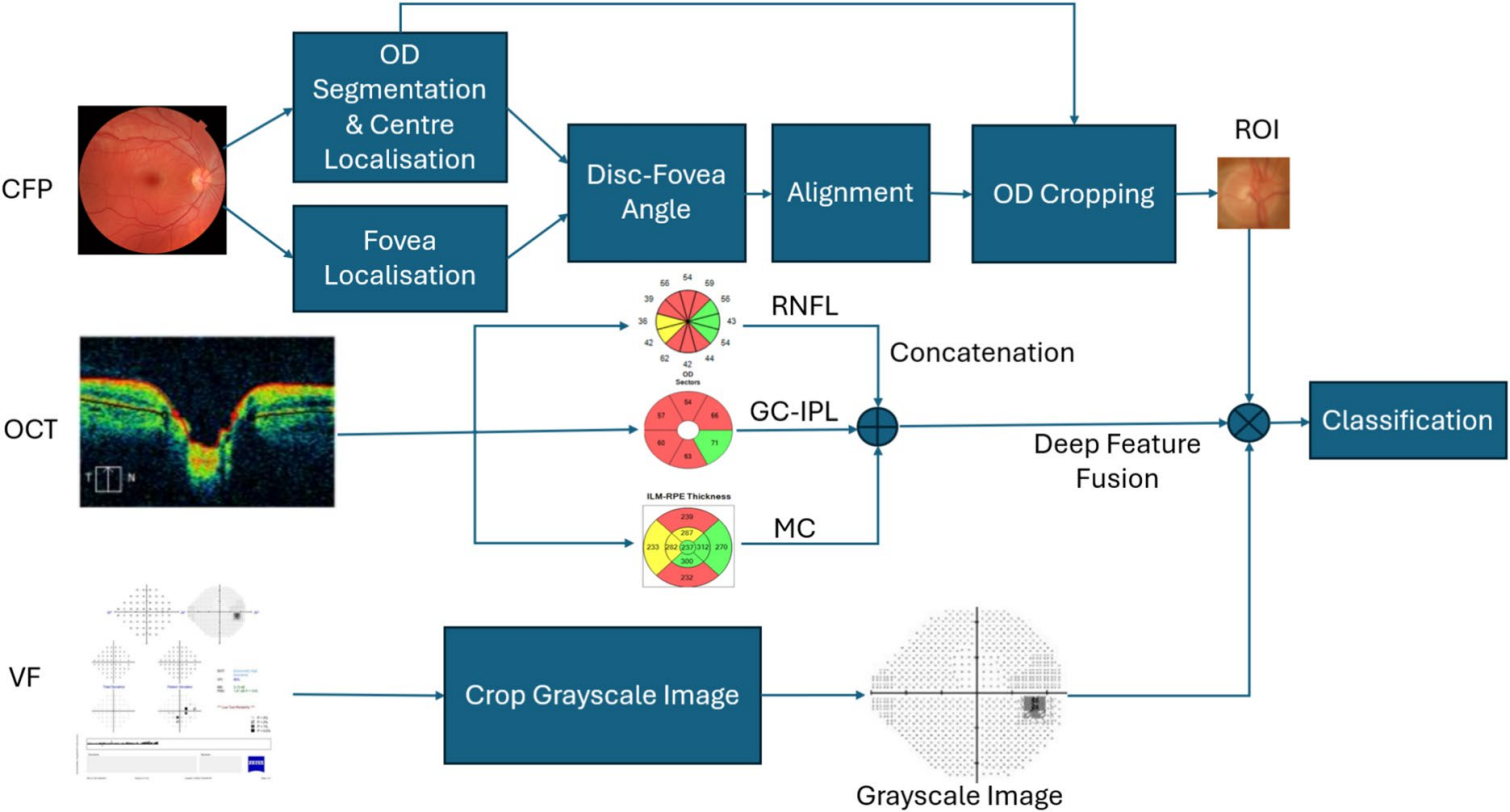
A high-level overview of the proposed multimodal feature fusion using visual field, colour fundus photographs and optical coherence tomography. *CFP* colour fundus photograph, *VF* visual fields, *OCT* optical coherence tomography, *OD* optic disc, *ROI* region of interest.

The method includes data acquisition and labelling, optic disc segmentation centre localisation, fovea localisation, disc-fovea alignment and optic disc cropping from modality-1 (CFPs), cropping of grayscale image from modality-2 (VFs), and finally deep intermediate level feature fusion from VF, CFPs and OCT modalities for deep multimodal classification (Fig. 1). The deep learning models were implemented using Python version 3.10.8 using TensorFlow, Keras and necessary libraries. The implemented codes in this study were run on an NVIDIA-SMI 560.28.03 Tesla V100-SXM2-32 GB GPU (CUDA Version: 12.6) using the computational cluster Katana supported by research technology services at UNSW Sydney.

### Data acquisition and labelling

Approval for ethical considerations was obtained from the University of New South Wales (HC210563), and all individuals provided written informed consent permitting the utilisation of their anonymised clinical information for research purposes. The study followed the principles of the Declaration of Helsinki. Data was acquired from patients who attended the Centre for Eye Health (CFEH) from 2015 to 2021. The diagnosis of glaucoma and other ocular conditions (including ION) followed CFEH procedures and protocols^38–40^, involving a thorough review of clinical data by a senior optometrist. Furthermore, an additional expert optometrist conducted a supplementary assessment to facilitate inclusion in the study. Among other neurodegenerative diseases, dementia and Parkinson’s patients were identified through self-reports and medication records in their medical histories. If a patient with a neurodegenerative disease was also found to have glaucoma, they were excluded from the cohort. Note that CFEH is a referral-only clinic, staffed by optometrist and consultant ophthalmologists with diagnosis and clinical audits also exist within the centre^34,35,41^. In this study, glaucoma patient’s ocular diagnoses were classified into three categories based on the mean deviation of visual fields (early glaucoma, moderate glaucoma, and advanced glaucoma), using criteria by Mills et al.^42^. Additionally, the central field defect was considered advanced although it does not meet the mean deviation criterion^43^. Healthy individuals were age-matched to neuropathy patients for reference labels in training a machine learning model (Table 1). Given that it is a multimodal study, both modalities were not present for a specific patient in some cases, which we have excluded from the final data preparation.

**Table 1:** Characteristics of the participants involved in this study.

Glaucoma types	N	Age (mean ± STD)	Right eye (RE)	Left Eye (LE)	RE + LE
Normal	228	68.2 ± 7.0	111	117	228
Early	69	65.2 ± 8.3	43	26	69
Moderate	60	64.6 ± 11.5	31	29	60
Advanced-MD	25	62.3 ± 11.5	19	6	25
Advanced (central VF defect)	59	60.9 ± 13.2	34	25	59
Glaucomatous (G) ON (total)	203	63.3 ± 11.1	127	86	213
Dementia	15	72.7 ± 9.1	15	15	30
Parkinson’s	15	66.3 ± 7.5	14	15	29
Ischaemic Optic Neuropathy (ION)	82	58.6 ± 12.9	41	41	82
Non-glaucomatous (NG) ON (total)	112	65.9 ± 9.8	70	71	141
Neuropathy = G + NG (total)	315	64.6 ± 10.5	197	157	354

All included patients had both CFP and VF available for the same eye.

### Optic disc segmentation and localisation

We utilised CFEH CFP data, a private dataset for neuropathy diagnosis. As an essential part of data preprocessing before the multimodal classification, the first step for multimodal analysis was to locate the optic disc (OD) centre of the CFEH images and calculate the disc-fovea angle and alignment^28^. Given that we did not have the ground truth localisation coordinates for our private CFEH dataset, we leveraged the use of transfer learning to locate the OD. The localisation was done using two methods: (i) considering it a regression problem and using EfficientNetB1^44^ as a feature extractor and (ii) semantic segmentation of the OD using U-Net^45^ with EfficientNetB1 as a backbone, and then identifying the centre of the OD. In both cases, the optic disc centre coordinates were predicted and located. For the regression problem, the IDRID dataset^46^ was used, and we considered another dataset named the ORIGA dataset^47^ for the OD segmentation task. The first approach was achieved by employing a fine-tuned EfficientNetB1^44^ network pre-trained on the ImageNet dataset^48^ for the OD localisation task (Supplementary Fig. 1). The second approach consisted of a U-Net architecture, utilizing the EfficientNetB1 model as the encoder^45^ for semantic segmentation and then localisation of the centre coordinates of the OD. Given that a U-Net network is composed of a symmetric structure with an encoder and a decoder, the encoder was based on EfficientNetB1^44^, leveraging pretrained weights from ImageNet^48^ to extract rich and hierarchical features from the input images (Supplementary Fig. 2).

### Fovea localisation

Fovea localisation was performed using both conventional computer vision techniques and a DL-based approach. The latter consisted of a similar architecture to EfficientNetB1^44^ as used for OD localisation. Then conventional computer vision-based approaches used three different methods for fovea localisation. The first method involved converting the original image to grayscale, extracting a circular region of interest (ROI) ${I}_{circ}(x,y)$ with r = r/3, applying Gaussian filtering^49^ and identifying the local minima^50^ (Supplementary Fig. 3). The second method comprised segmenting the ROI to superpixels using Simple Linear Iterative Clustering (SLIC) superpixel segmentation^51^ and identifying the darkest pixel within the darkest superpixels. The third method was a combination of the previous two methods, i.e. applying Gaussian filtering and identifying local minima in the darkest superpixel within the ROI to determine the foveal location. (Supplementary Algorithm 1).

### Disc fovea alignment and cropping

Once the OD segmentation-localisation network was trained and the fovea localisation method was trained and tested with the public datasets, the models were used to locate OD and fovea from the CFEH private dataset. Once the OD and fovea were located, the disc-fovea angle was measured and thus the image was rotated accordingly so the disc and fovea align. After alignment, images were supplied again to the Efficient-UNet segmentation model to crop the optic disc. On the other hand, the VF grayscale images were cropped from raw visual field images using the specific coordinates of the top left and bottom right coordinates.

### Deep unimodal and multimodal classification

Diagnosis of glaucoma and neurodegenerative disease was performed utilising both unimodal and multimodal approaches with a two-tiered classification system—using a top-level (or primary) classifier and a sub-level (or secondary) classifier. The primary classifier first distinguished between normal participants and Optic Neuropathy (ON) cases, with ON encompassing both glaucomatous ON and non-glaucomatous ON conditions, including dementia, Parkinson’s disease and ION. The secondary classifier then focused on cases identified as ON by the primary classifier, further distinguishing glaucoma from other non-glaucoma conditions, including dementia, Parkinson’s disease and ION.

The unimodal approaches were developed separately for CFP and VF grayscale images, using a baseline convolutional neural network (CNN) and state-of-the-art CNN architectures VGG-16^52^, Mobilenet-v1^53^, LeNet-5^54^, and Vision Transformer (ViT-16^55^). Among these, VGG and MobileNet facilitate hierarchical feature representation and transfer learning using pre-trained weights with the ImageNet dataset^48^. ViT-16 leverages self-attention mechanisms to model long-range dependencies within images, enabling effective feature learning without relying on convolutional operations^9^.

For multimodal classification, the cropped OD using the segmentation model and the cropped grayscale VF images were supplied to a custom CNN for feature extraction and the deep feature fusion was performed using multiple approaches: (i) custom CNN followed by concatenation, (ii) passing the CNN extracted features to a spatial-attention module followed by concatenation, (iii) passing the CNN extracted features to a spatial-attention module and then self-modal attention module followed by concatenation, and (iv) passing the CNN extracted features to a spatial attention module, then a self-attention module and finally to a cross-modal attention module before the features were concatenated. All classification models were evaluated with fivefold cross-validation with patient level splitting from the CFEH dataset, so each pair of eyes remains in either in training or test set.

### Base models without attention

In both unimodal and multimodal models without attention, a CNN architecture was used to extract features, followed by a fully connected layer for classification (Supplementary Fig. 4). In the uni- and multimodal models, the features extracted by the deep CNN from each modality were represented as tensors. We also used shallow state-of-the-art ImageNet-based CNN network architectures (fine-tuned VGG-16 and MobileNet-v1, LeNet-5), and a Siamese Network with L1-distance and Cross-Entropy Loss. In addition to the base model without attention, we have used a transformer-based architecture (ViT-16), which inherently employs a multi-head self-attention mechanism.

### Multimodal models with attention mechanism

**Fig. 2:**
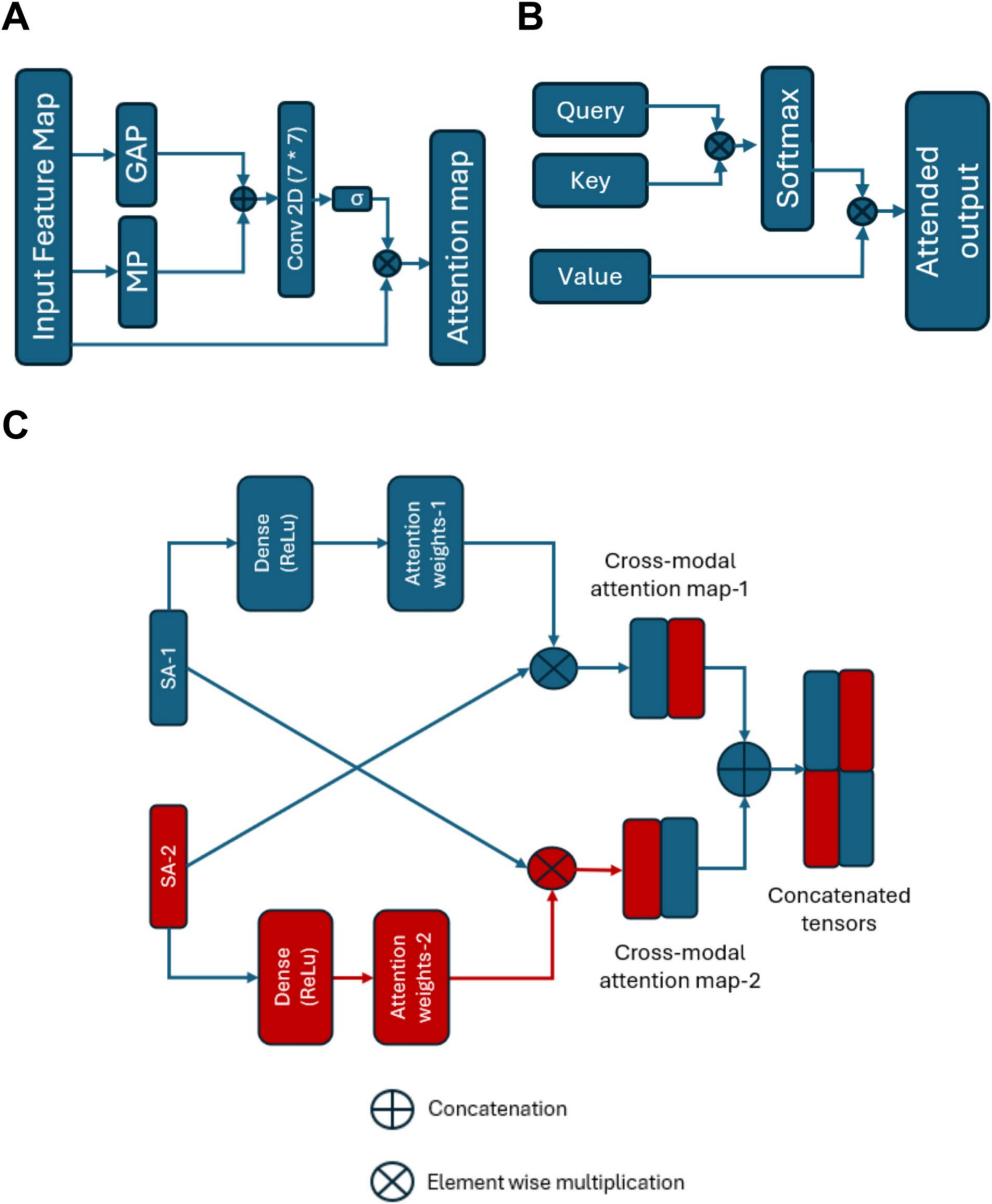
Different attention mechanisms. (**A**) Spatial attention module (S-module). (**B**) Self-attention module (I-module). (**C**) Cross-modal attention module (C-module) with concatenation at the end. Note: σ denotes the sigmoid function, SA-1; Spatial attention map modality-1, SA-2; Spatial attention map modality-2. The developed models plugged in singular and stacked attention modules (S module, S-I module and S-I-C module).

The attention mechanism, originally introduced by Bahdanau et al.^56^ and further refined by Vaswani et al.^33^, has significantly impacted computer vision by enabling models to dynamically focus on the most relevant parts of an image, thus enhancing performance in tasks like image classification. By integrating different attention mechanisms with CNNs (e.g. VGG), these models become more efficient and interpretable, capturing long-range dependencies and highlighting influential input regions. In this study, we have used three attention mechanisms: spatial attention (intramodal attention), self-attention (intramodal attention), and cross-modal attention (intermodal attention). First, the spatial attention mechanism selectively focuses on relevant spatial regions within the input feature maps of each modality^57^ (Fig. 2A).

1 $$SA\left(F\right)=\sigma \left({Conv f}^{7\times 7}\left(\left[AvgPool \left(F\right);MaxPool \left(F\right)\right]\right)\right)\odot F .$$

Here, $F$ is the input feature map, $AvgPool$ is the average pooling operation, $MaxPool$ is the max pooling operation, $Conv {f}^{7\times 7}$ is the convolution operation with a 7 × 7 filter with a sigmoid activation, $\sigma$ denotes sigmoid function and ⊙ denotes elementwise multiplication.

Second, following the spatial attention module applied to each modality, we introduced a self-attention mechanism to enhance the representation of features by capturing long-range dependencies and relationships across different spatial locations^33^ (Fig. 2B). This mechanism aids in refining the spatially attended features obtained earlier, ensuring that the most relevant information contributes prominently to the subsequent fusion process.

$$SelfAttn \left(X\right)=Softmax(\frac{\mathrm{Q} { \left(\mathrm{K}\right)}^{T}}{\sqrt{{d}_{k}}}) \mathrm{V}$$

2 $$=Softmax\left(\frac{{(XW}^{Q}) { \left({XW}^{K}\right)}^{T}}{\sqrt{{d}_{k}}}\right){XW}^{V}.$$

Here, X is the input feature map, Q= ${W}^{Q}$ X, K= ${W}^{K}$ X, V= ${W}^{V}$ X are the query, key, and value matrices, where the ${W}^{Q}$, ${W}^{K}$, ${W}^{V}$ are learned weight matrices, ${d}_{k}$ is the dimensionality of the key vector and the SoftMax function is applied to the scaled dot-product of the query and key to compute the attention weights.

Third, following the self-attention module, we used a cross-modal attention mechanism to facilitate the effective cross-integration of information across the two modalities^58^, achieved through element-wise multiplication of attended features (Fig. 2C).

3 $$CMA\left({(X}_{1}, {X}_{2}\right)=\left( {(X}_{1}\odot {{ReLu (W}_{1}X}_{2}); {X}_{2}\odot {{ReLU (W}_{2}X}_{1})\right).$$

**Fig. 3:**
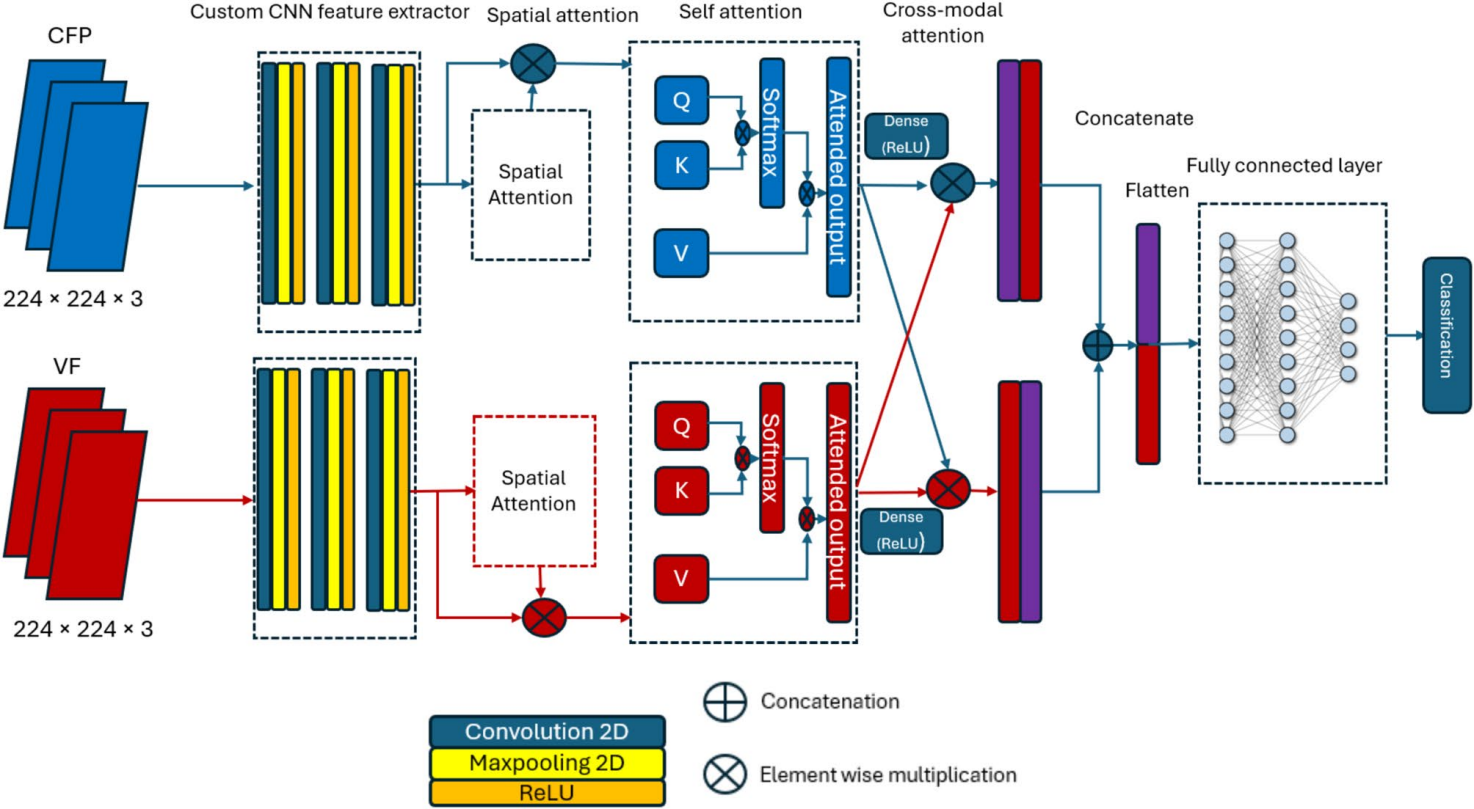
Multi-attention-based deep multimodal fusion network (MAM-Fusion-Net) for neuropathy diagnosis using colour fundus photographs and visual fields. The CNN-extracted features are passed through the Spatial + Self + Cross-modal attention module (S-I-C module). CFP: Colour Fundus Photograph, VF: Visual Fields.

Here, ${X}_{1}$ and ${X}_{2}$ are the two input feature maps from the two different modalities, ${W}_{1}$ and ${W}_{2}$ are the learned weight metrics, and $ReLU$ denotes the activation function. In this study, the three attention modules and their combinations were considered:Spatial attention module (S module), the Spatial + Self-attention module (S-I module), and the Spatial + Self + Cross-modal attention module (S-I-C module, Fig. 3).

### OCT-based feature extraction and classification

Alongside VF and CFP images, we extracted features from OCT scans, focusing on 45 spatial domain parameters including RNFL, ganglion cell-inner plexiform layer (GC-IPL), and macular (MC) thickness. These features (Supplementary Table 1) provide valuable insight into retinal layer distribution and structural variations, which are clinically significant for glaucoma diagnosis and staging, as identified by explainable machine learning^34,35^. CIRRUS HD-OCT software was used to extract these numerical features for further analysis.

Given the computational complexity and cost associated with different models as highlighted in previous studies^59–61^, we employed four supervised learning algorithms for classification using the OCT features for unimodal analysis: K-Nearest Neighbour (KNN), Support Vector Machines (SVM), Random Forests (RF), and Multi-Layer Perceptron (MLP). Each classifier’s hyperparameters were optimised iteratively to achieve the highest accuracy through cross-validation. For the KNN classifier, a range of values for 'k' (1 ≤ k ≤ 40) was tested, with the optimal 'k' selected based on training accuracy. The SVM model utilised a Gaussian radial basis function (RBF) kernel, with the regularisation parameter 'C' and kernel coefficient 'γ' fine-tuned using grid-search. In the RF classifier, the number of estimators (‘n_estimators’) and the maximum tree depth (‘max_depth’) were optimised via grid search^35,59–61^. For the MLP classifier, a feedforward neural network was considered with the number of hidden layers, optimiser, activation function and learning rates fine-tuned to achieve optimal performance. When OCT was fused with CFPs and VFs in the deep fusion stage, the handcrafted features were combined with the cross-modal attended output from CFP-VF fusion before they were flattened and passed through the fully connected layer for classification (Supplementary Fig. 5).

### Classification performance evaluation metrics

The effectiveness of the machine learning classifiers was assessed using sensitivity (true positive rate, TPR), specificity (true negative rate, TNR), accuracy, and F1-score, as defined in Eqs. (4)−(7). These metrics were computed from the confusion matrix, which comprises true positive (TP), true negative (TN), false positive (FP), and false negative (FN) values. In the context of neuropathy diagnosis, TP represents correctly identified neuropathy cases, whereas FP denotes cases incorrectly classified as neuropathy. Conversely, TN corresponds to correctly identified non-neuropathy cases, while FN represents cases that were incorrectly classified as non-neuropathy despite having the condition. The area under the receiver operating characteristic (ROC) curve (AUC), which quantifies the classifier’s ability to distinguish between classes, along with sensitivity, specificity, F1-score, and accuracy, was reported to evaluate the model’s performance.

4 $$\text{Sensitivity or TPR or Recall}= \frac{\mathrm{TP}}{\mathrm{TP}+\mathrm{FN}} \times 100\mathrm{\%},$$

5 $$\text{Specificity or TNR}= \frac{\mathrm{TN}}{\mathrm{TN}+\mathrm{FP}} \times 100\mathrm{\%},$$

6 $$\mathrm{Accuracy}= \frac{\mathrm{TP}+\text{ TN}}{\mathrm{TP}+\mathrm{TN}+\mathrm{FP}+\mathrm{FN}} \times 100\%,$$

7 $$\mathrm{F}1-\text{Score }= \frac{2 \times \text{ Precision }\times \text{ Recall}}{\mathrm{Precision}+\text{ Recall}} \times 100\%.$$

## Results

### Optic disc segmentation and localisation

**Fig. 4:**
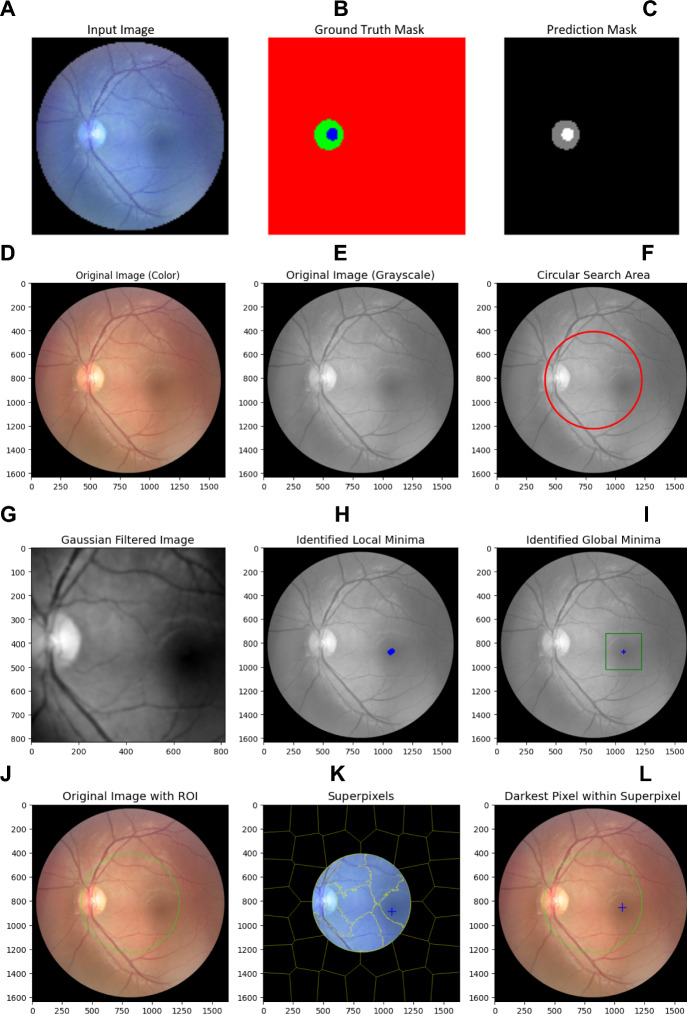
Optic disc and fovea localisation example. Optic disc localisation (**A** − **C**). (**A**) Example input image, (**B**) ground truth mask, and (**C**) predicted mask. Different steps of fovea localisation using Gaussian filtering and identifying local minima (**D** − **I**). (**D**) Original colour image. (**E**) Grayscale image. (**F**) Circular search area. (**G**) Gaussian filtered image. (**H**) Identified local minima. (**I**) Identified global minima (taking the mean of the coordinates of the identified local minima, where there are multiple local minima). Different steps of fovea localisation by using superpixeling (**J** − **L**). (**J**) Original image with the circular search area. (**K**) Superpixels within the circular search area. (**L**) Identification of the darkest pixel within the darkest superpixel.

The first approach for OD localisation was a fine-tuned EfficientNetB1 network as a regressor to predict the OD centre coordinates. This network was trained and tested on the IDRID dataset^62^, resulting in an Intersection over Union (IoU) of 0.870 (Standard Error, SE: 0.009) and mean Euclidean Distance (ED) of 4.663 (SE: 0.3360) (scaled to %, considering the different size of the image in different dataset) between the actual and predicted coordinates (Table 2). The second approach, which includes the EfficientNetB1 as a backbone to UNet architecture for semantic segmentation, was trained on the ORIGA dataset^47^ (given the availability of the segmentation masks (Fig. 4A−C)). For segmentation performance, it was tested on the same dataset and for localisation it was tested on the IDRID dataset (given the availability of actual coordinates). For the OD and cup semantic segmentation with the Efficient-UNet, it produced a sensitivity of 0.998, accuracy of 0.998, AUC of 0.999, F1-score of 0.953, and Dice score of 0.955. The segmentation-based model produces a superior performance to the sole EfficientNetB1-based regression model in OD localisation, with an IoU of 0.980 (SE: 0.001) and mean ED of 0.673 (SE: 0.030) (%) (Table 2). Based on the superior performance of the later model, it was used for both localisation on our private CFEH dataset for OD localisation and alignment with fovea, and later OD cropping.

**Table 2:** Performance of optic disc and fovea localisation methods.

Task	Model/method	Train dataset	Test dataset	IoU (mean ± SE)	ED (related to original dimension) (mean ± SE)	ED Scaled (scaled to %) (mean ± SE)
OD centre localisation	EfficientNetB1 (as feature extractor)	IDRID (413)	IDRID (103)	0.870 ± 0.009	166.370 ± 11.980	4.663 ± 0.336
**OD segmentation & centre localisation**	**UNet (EfficientNetB1 as backbone)**	ORIGA (520)	IDRID (103)	**0.980 ± 0.001** (localisation)	**24.000 ± 1.060**	**0.673 ± 0.030**
Fovea localisation	EfficientNetB1 (as feature extractor)	REFUGE2 (320)	REFUGE2 (80)	0.860 ± 0.007	131.060 ± 7.840	6.271 ± 0.375
	**Gaussian filtering**	−	REFUGE2 (80)	0.920 ± **0.005**	72.190 ± **6.570**	3.454 ± **0.314**
	Superpixel	−	REFUGE2 (80)	0.940 ± 0.014	55.880 ± 15.450	2.674 ± 0.739
	Superpixel + Gaussian	−	REFUGE2 (80)	**0.950** ± 0.011	**52.020** ± 12.600	**2.489** ± 0.603

The ED was scaled (to %) as presented in the last column, considering the different dimensions of the images in different datasets. $Percentage distance=\frac{ED}{Image size}\times 100\%. Image size=\frac{Width+Height}{2}$. Significant values are in bold.

### Fovea localisation

The first approach utilised for fovea localisation was a deep learning approach, similar to OD localisation based on EfficientNetB1 as a regressor. The EfficientNetB1 was trained (320 training images) and tested (80 test images) on the REFUGE2 dataset^63^ (based on the availability of fovea coordinates), which produced a mean IoU of 0.86 (SE: 0.007) and ED of 6.271 (SE: 0.375) (Table 2). The other three approaches used for fovea localisation use traditional image processing-based approaches using the same test images from the REFUGE2 dataset. The first two traditional approaches using Gaussian filtering (Fig. 4D–I) and superpixeling (Fig. 4J−L) separately to predict fovea location produced an IoU of 0.920 (SE: 0.005) and 0.940 (SE: 0.014), and ED of 3.454 (SE: 0.314) and 2.674 (SE: 0.739), respectively.

Between these two approaches, the Gaussian filtering-based approach produced slightly lower performance (IoU: 0.920) than superpixeling in terms of mean value, but the standard error was lowest (0.005). When a combination of the two approaches was used, i.e. superpixeling and applying Gaussian filtering in the darkest superpixel to identify local minima, this method produced better performance with relatively lower SE compared to the sole superpixeling approach, having an IoU of 0.950 (SE:0.011) and ED of 2.489 (SE:0.603). However, the simpler approach using Gaussian filtering has low variability (SE:0.005) and lower computational cost, therefore, it was finally used on the CFEH private data for fovea localisation and alignment with OD.

### Deep classification using unimodal models

The deep multimodal analysis was performed with sole CNN-based and CNN plus attention-based approaches. All the unimodal and multimodal models were trained, validated and tested using a fivefold cross-validation, with sensitivity, specificity, accuracy, F1-score and area under the receiver operating characteristic curve (AUC) being the performance metrics (Table 3, Supplementary Table 2). The number of total, trainable and non-trainable parameters was also noted for each trained model (Supplementary Table 3).

**Table 3:** Performance of the classification using unimodal and multimodal approaches for neuropathy (glaucoma, ischaemic optic neuropathy, dementia and Parkinson’s disease) vs normal classification.

Attention (Fusion type)		Model	Mean ± SD					95% CI				
			Sensitivity	Specificity	Accuracy	AUC	F1-score	Sensitivity	Specificity	Accuracy	AUC	F1-score
Unimodal	**CFP**	CNN	0.667 ± 0.050	0.780 ± 0.092	0.721 ± 0.053	0.807 ± 0.062	0.713 ± 0.041	0.623–0.711	0.699–0.861	0.675–0.767	0.753–0.861	0.677–0.749
		VGG-16	**0.831 ± 0.043**	0.716 ± 0.092	**0.776 ± 0.039**	**0.846 ± 0.049**	**0.793 ± 0.042**	0.793–0.869	0.635–0.797	0.742–0.810	0.803–0.889	0.756–0.830
		MobileNet-v1	0.787 ± 0.111	0.576 ± 0.124	0.681 ± 0.056	0.761 ± 0.083	0.717 ± 0.058	0.690–0.884	0.467–0.685	0.632–0.730	0.688–0.834	0.666–0.768
		LeNet-5	0.812 ± 0.048	0.677 ± 0.103	0.748 ± 0.055	0.816 ± 0.040	0.771 ± 0.044	0.770–0.854	0.587–0.767	0.700–0.796	0.781–0.851	0.732–0.810
		ViT-16	0.748 ± 0.102	**0.803 ± 0.123**	0.776 ± 0.097	0.840 ± 0.068	0.774 ± 0.101	0.658–0.837	0.695–0.911	0.691–0.861	0.781–0.900	0.685–0.862
	**VF**	CNN	0.679 ± 0.116	**0.844 ± 0.094**	0.787 ± 0.077	0.835 ± 0.089	0.764 ± 0.092	0.577–0.781	0.762–0.926	0.720–0.854	0.757–0.913	0.683–0.845
		VGG-16	**0.859 ± 0.100**	0.731 ± 0.229	0.795 ± 0.088	**0.873 ± 0.058**	0.815 ± 0.069	0.771–0.947	0.530–0.932	0.718–0.872	0.822–0.924	0.755–0.875
		MobileNet-v1	0.842 ± 0.050	0.746 ± 0.241	**0.795 ± 0.121**	0.860 ± 0.099	**0.816 ± 0.087**	0.798–0.886	0.535–0.957	0.689–0.901	0.773–0.947	0.740–0.892
		LeNet-5	0.748 ± 0.127	0.736 ± 0.140	0.736 ± 0.053	0.865 ± 0.062	0.743 ± 0.059	0.637–0.859	0.613–0.859	0.690–0.782	0.811–0.919	0.691–0.795
		ViT-16	0.769 ± 0.107	0.729 ± 0.134	0.747 ± 0.100	0.831 ± 0.091	0.758 ± 0.095	0.675–0.863	0.612–0.847	0.659–0.834	0.752–0.911	0.674–0.841
	**OCT**	KNN	0.769 ± 0.107	0.729 ± 0.134	0.747 ± 0.100	0.831 ± 0.091	0.758 ± 0.095	0.767–0.886	0.870–0.955	0.825–0.912	0.827–0.912	0.819–0.912
		SVM	**0.827 ± 0.068**	**0.912 ± 0.049**	**0.868 ± 0.050**	0.869 ± 0.049	**0.865 ± 0.053**	0.701–0.870	0.790–0.903	0.769–0.860	0.861–0.917	0.759–0.864
		RF	0.785 ± 0.096	0.847 ± 0.065	0.815 ± 0.052	0.889 ± 0.032	0.812 ± 0.060	0.758–0.887	0.795–0.907	0.794–0.878	0.848–0.937	0.793–0.881
		MLP	0.823 ± 0.074	0.851 ± 0.064	0.836 ± 0.048	**0.892 ± 0.051**	0.837 ± 0.050	0.749–0.921	0.693–0.826	0.736–0.860	0.855–0.921	0.745–0.871
Multimodal-Dual	**CFP + VF** (no attention)	CNN	0.749 ± 0.141	0.862 ± 0.114	0.799 ± 0.062	0.903 ± 0.046	0.791 ± 0.074	0.625–0.873	0.762–0.962	0.745–0.853	0.863–0.943	0.726–0.856
		VGG-16	0.806 ± 0.086	0.803 ± 0.111	0.804 ± 0.084	0.899 ± 0.052	0.810 ± 0.079	0.731–0.881	0.706–0.900	0.730–0.878	0.853–0.945	0.741–0.879
		MobileNet-v1	0.898 ± 0.059	0.814 ± 0.132	0.858 ± 0.041	0.885 ± 0.040	0.870 ± 0.026	0.846–0.950	0.698–0.930	0.822–0.894	0.850–0.920	0.847–0.893
		LeNet-5	0.889 ± 0.116	0.717 ± 0.113	0.804 ± 0.035	0.891 ± 0.040	0.822 ± 0.038	0.787–0.991	0.618–0.816	0.773–0.835	0.856–0.926	0.789–0.855
		Siamese	0.572 ± 0.524	0.641 ± 0.414	0.611 ± 0.147	0.756 ± 0.062	0.462 ± 0.426	0.113–1.000	0.278–1.000	0.482–0.740	0.702–0.810	0.089–0.835
	**CFP + VF** (ViT-based multi-head self-attention)	ViT-16	0.877 ± 0.056	0.872 ± 0.086	0.876 ± 0.063	0.928 ± 0.042	0.880 ± 0.058	0.828–0.927	0.797–0.948	0.821–0.930	0.891–0.965	0.829–0.930
	**CFP + VF** (Single attention)	CNN + S	0.805 ± 0.105	0.931 ± 0.030	0.821 ± 0.064	0.930 ± 0.049	0.802 ± 0.079	0.713–0.897	0.905–0.957	0.765–0.877	0.887–0.973	0.733–0.871
	**CFP + VF** (Dual attention)	CNN + S-I	0.854 ± 0.055	0.925 ± 0.062	0.888 ± 0.046	0.955 ± 0.030	0.888 ± 0.043	0.806–0.902	0.871–0.979	0.848–0.928	0.929–0.981	0.850–0.926
	**CFP + VF** (Triple attention)	**CNN + S-I-C [MAM-Fusion-Net]**	**0.919 ± 0.044**	**0.964 ± 0.031**	**0.941 ± 0.034**	**0.981 ± 0.020**	**0.942 ± 0.032**	0.880–0.958	0.937–0.991	0.911–0.971	0.963–0.999	0.914–0.970
		VGG-16 + S-I-C	0.863 ± 0.058	0.938 ± 0.029	0.899 ± 0.042	0.960 ± 0.025	0.898 ± 0.042	0.812–0.914	0.913–0.963	0.862–0.936	0.938–0.982	0.861–0.935
		MobileNet-v1 + S-I-C	0.939 ± 0.036	0.912 ± 0.032	0.926 ± 0.031	0.956 ± 0.037	0.929 ± 0.029	0.907–0.971	0.884–0.940	0.899–0.953	0.924–0.988	0.904–0.954
		LeNet-5 + S-I-C	0.856 ± 0.062	0.862 ± 0.091	0.861 ± 0.064	0.929 ± 0.070	0.864 ± 0.060	0.802–0.910	0.782–0.942	0.805–0.917	0.868–0.990	0.811–0.917
		Siamese + S-I-C	0.869 ± 0.060	0.876 ± 0.048	0.873 ± 0.027	0.928 ± 0.060	0.876 ± 0.030	0.816–0.922	0.834–0.918	0.849–0.897	0.875–0.981	0.850–0.902
		ViT-16 + S-I-C	**0.919 ± 0.105**	0.920 ± 0.148	0.920 ± 0.066	0.955 ± 0.025	0.923 ± 0.059	0.828–1.000	0.790–1.000	0.862–0.977	0.933–0.977	0.872–0.975
Multimodal**-**Triple	**CFP + VF + OCT** (Triple attention)	**CNN + S-I-C [MAM-Fusion-Net] plus OCT**	**0.970 ± 0.036**	**0.987 ± 0.019**	**0.979 ± 0.02**	**0.998 ± 0.002**	**0.978 ± 0.021**	0.938–1.000	0.970–1.000	0.961–0.996	0.996–1.000	0.960–0.997

Bolded numbers represent the highest performance within the unimodal and multimodal approaches. S: Spatial attention module; S-I: Spatial-Self attention module, S-I-C: Spatial-Self-Cross-modal-attention module; All VGG-16/MobileNet-v1 models used ‘ImageNet’ pretrained weights. Without ‘ImageNet’, weights, the model was overfitting. All multimodal models went through concatenation at the end. *KNN* K-nearest neighbours, *SVM* support vector machine, *RF* random forest, *MLP* multilayer perceptron, *CFP* colour fundus photographs, *VF* visual fields, *OCT* optical coherence tomography.

### Deep unimodal models using CFP

Among the unimodal approaches, a baseline CNN was applied on CFPs for neuropathy diagnosis, which produced a sensitivity of 0.667, specificity of 0.780, accuracy of 0.721 and F1-score of 0.713, covering an area under the ROC curve of 0.807 (95% CI 0.753–0.861) (Table 3). A fined-tuned VGG16 network (first 6 layers) and MobileNet-v1 (first 6 layers) with ImageNet weights, LeNet-5 and ViT-16 were also applied in place of simple CNN architecture. Using the CFPs the state-of-the-art models produced a sensitivity in the range of 0.787–0.831, specificity of 0.576–0.716, and accuracy metrics within a range of 0.681–0.776, F1-score between 0.717–0.793, with an AUC of 0.761–0.846, with the fine-tuned VGG-16 model producing superior performance than the other models in terms of AUC.

### Deep unimodal models using VFs

When the baseline CNN was applied to VFs for neuropathy diagnosis, it produced comparatively better performance than the CFPs, with a sensitivity of 67.9%, specificity of 84.4%, accuracy of 78.7% and F1-score of 76.4%, covering an area under the ROC curve of 0.835 (95% CI 0.757–0.913) (Table 3). On the other hand, using the VFs the state-of-the-art models produced comparatively higher sensitivity in the range of 74.8–85.9%, specificity of 73.1–74.6%, and accuracy metrics within a range of 73.6–79.5%, F1-score between 74.3–81.6%, with an AUC of 0.860–0.865, with the VGG-16 model producing superior performance than the other models in terms of AUC. In short, the unimodal models using state-of-the-art models, especially models with ImageNet weights (VGG-16 and MobileNet-v1) produce superior performance than the simple custom CNN model and ViT-16. However, the number of trainable parameters was substantially higher in the state-of-the-art models (range 0.08–85.82 million (M)) compared to the custom CNN (0.03 M) (Supplementary Table 3).

### Traditional unimodal models using OCT

Using the traditional machine learning models with the OCT-extracted features produced comparable and relatively better performance than the CFP/VF-based unimodal deep learning models. The four classical classifiers produced a sensitivity ranging from 0.769–0.827, a specificity ranging 0.729–0.912, an accuracy ranging 0.747–0.868, F-1 score ranging 0.758–0.865. SVM generates the highest sensitivity of 0.827, specialty of 0.912, accuracy of 0.868 and F1-score of 0.865. In terms of AUC, MLP performed the best, covering an area of 0.892, while KNN was the least performing classifier with AUC score of 0.831. The performance of the OCT-extracted features using traditional classifiers shows the utility of RNFL, GC-IPL and MC thickness features in neuropathy diagnosis.

### Deep classification using multimodal models (CFPs + VFs)

The deep multimodal network architectures were trained and tested using two approaches: with and without attention mechanisms. In the attention-based networks, single, dual, and triple attention modules were applied. Initially, the custom CNN was used without any attention (only employing concatenation), followed by versions with attention mechanisms: a spatial attention module (S-module), a spatial and self-attention module (S-I module), and finally, a combination of spatial, self, and cross-modal attention (S-I-C module). Later, the custom CNN was replaced with state-of-the-art networks including a fine-tuned VGG-16, MobileNet-v1 (using ImageNet weights), LeNet-5, a Siamese CNN network with L1 distances and ViT-16. All multimodal networks incorporated concatenation at the final stage to fuse extracted feature maps from the two modalities into a unified representation.

### Multimodal model without attention

The custom CNN model with concatenation produces a mean sensitivity of 0.749, specificity of 0.862, accuracy of 0.799, F1-score of 0.791, having 0.903 AUC (95% CI 0.863–0.943) across a fivefold cross-validation (Table 3). When the CNN was replaced by a fine-tuned VGG16 (with pre-trained ImageNet weights), MobileNet-v1 (with pre-trained ImageNet weights), and LeNet-5 network, it produced a mean sensitivity ranging from 0.806–0.898, specificity of 0.717–0.814, accuracy of 0.804–0.858, F1-score of 0.810–0.870, having 0.891–0.903 AUC. While the fine-tuned VGG-16 (using the first six convolutional layers), MobileNet-v1 (six convolutional layers) and LeNet-5 produced an AUC above 0.880, the Siamese network produced the lowest performance (AUC: 0.756). The number of trainable parameters was 0.06 M for CNN, MobileNet-v1 and Siamese models, with the highest number of parameters for VGG-16 and LeNet-5 (0.90 million (M)).

### Multimodal model with attention mechanism

**Fig. 5:**
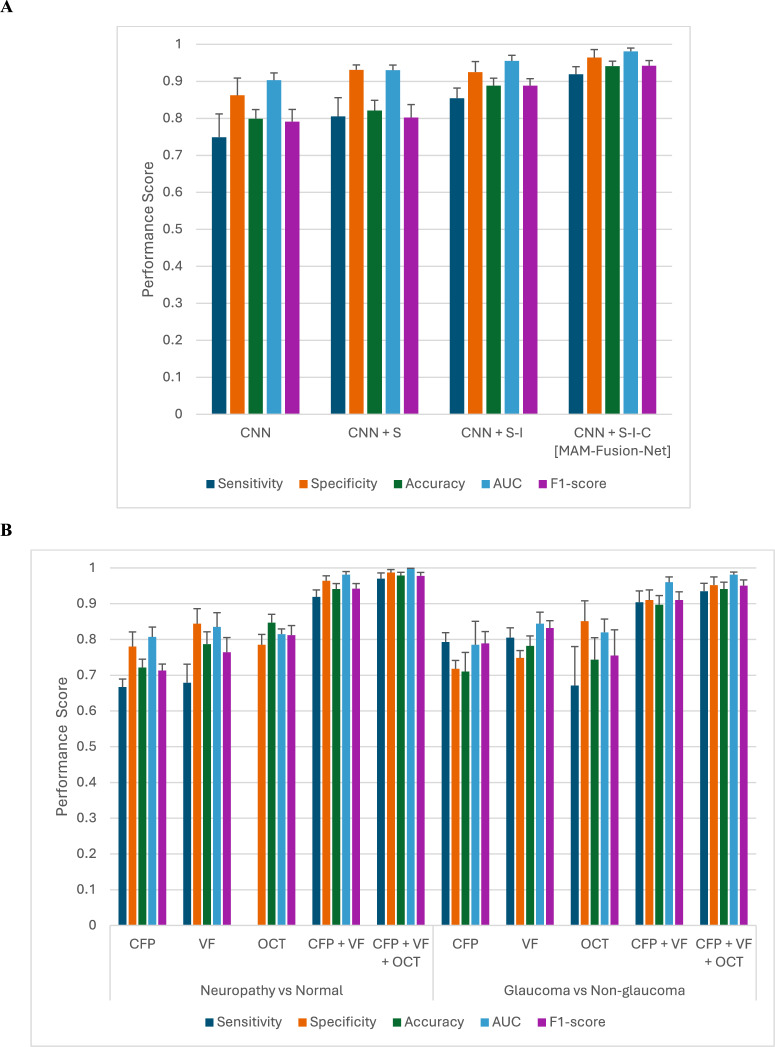
Bar plots of obtained performance measures. (**A**) For optic neuropathy (glaucoma, ischaemic optic neuropathy, dementia and Parkinson’s disease) versus normal participant diagnosis using the top-level classifiers, a comparison of custom CNN, CNN + S, CNN + S-I, CNN + S-I-C (MAM-Fusion-Net) models using CFPs and VFs. (**B**) Comparison of unimodal (CFP, VF) and multimodal (CFP + VF, CFP + VF + OCT) performance for top-level (neuropathy vs normal participants) and sub-level (glaucoma vs non-glaucoma) classifiers. Reported CFP/VF-based unimodal models use custom CNN, OCT-based unimodal performance was reported based on SVM, while multimodal models use MAM-Fusion-Net. The error bars represent standard error across the 5-folds (*SE* standard deviation/$\sqrt{5}$). *CFP* colour fundus photographs, *VF* visual fields, *OCT* optical coherence tomography S: Spatial attention module; S-I: Spatial-Self attention module, S-I-C: Spatial-Self-Cross-modal-attention module.

Adding a spatial attention module (S-module) with the CNN improved the AUC slightly from 0.903 (95% CI 0.863–0.943) to 0.930 (95% CI 0.887–0.973). Joining a self-attention module after the spatial modal attention module improves it substantially to 0.955 (95% CI 0.929–0.981). The addition of the third mechanism cross-modal attention module after the spatial-self attention (S-I) module creates a spatial-self-cross modal attention module (S-I-C module), and the generated model multi-attention-multimodal-network (MAM-Fusion-Net) produces a superior performance than the others (S or S-I module), with an AUC of 0.981 with lowest standard deviation (SD: 0.020, 95% CI 0.963–0.999). It produces a sensitivity, specificity, accuracy and F1-score of 0.919 (95% CI 0.880–0.958), 0.964 (95% CI 0.937–0.991) and 0.941 (95% CI 0.911–0.971), and 0.942 (95% CI 0.914–0.970) with the lowest SD compared to the previous mechanisms (Fig. 5). When the CNN was replaced by a fine-tuned (first six convolutional layers) VGG-16 and MobileNet-v1 with pre-trained ImageNet weights, LeNet-5 and Siamese network, it produced a mean sensitivity ranging from 0.856–0.939, specificity of 0.862–0.938, accuracy of 0.861–0.926, F1-score of 0.864–0.929, having 0.929–0.960 AUC. The ViT-16 model produced a sensitivity of 0.877, specificity of 0.872, accuracy of 0.976, F1- score of 0.880 and an AUC score of 0.928. When the S-I-C module was added to it at the end, the performance increased to 0.919 sensitivity, 0.920 specificity, 0.920 accuracy, 0.923 F-1 score and 0.955 AUC score.

A two-tailed paired t-test was performed to compare the performance of the models without and with attention mechanism across fivefold cross-validation (Supplementary Table 4). The t-test between the CNN and CNN + S modules shows a non-significant difference; however, adding the self-attention module on top of the spatial attention produces significant differences (p < 0.05, between CNN and CNN + S-I module) in terms of accuracy, AUC and F1-score. When the cross-modal attention mechanism was introduced on top of the S-I module, it showed highly significant (p < 0.001, between CNN and CNN + S-I-C module) accuracy, with very significant (p < 0.01) AUC and F-1 scores. For the state-of-the-art models with and without attention mechanism, a significant (p < 0.05) increase is observed in accuracy, AUC and F1-score for VGG-16 with S-I-C module compared to the sole VGG -16 model without attention modules. For the MobileNet-v1 model, the difference is very significant (p < 0.01) in terms of accuracy, AUC and F1 score. This implies that using the S-I-C module with the lightweight model with ImageNet weights boosts the performance of multimodal fusion. A t-test between the ViT-16 with and without the S-I-C module showed a non-significant increase. This is probably due to the fact that ViT-16 already uses a multi-head self-attention mechanism, which captures the modality specific features.

Incorporating the spatial attention module in the CNN increased the trainable parameters to 2.99 M. However, introducing the self-attention mechanism (S-I module) effectively halved the parameters to 1.51 M, with only a slight increase when the cross-modal attention (S-I-C module) was added, keeping the count at 1.51 M. This reduction occurs because the self-attention layers output fewer channels (16) (third dimension of 3D tensors) compared to the spatial-only model (32), leading to smaller input dimensions for subsequent concatenation and dense layers, thereby reducing the overall parameter count. Compared to state-of-the-art models like LeNet-5 (5.27 M), MobileNet-v1 (7.62 M), VGG-16 (10.04 M), Siamese models with attention (16.64 M) and ViT-16 (85.82 M), our custom CNN with the S-I-C module (MAM-Fusion-Net) achieves high performance with a lightweight design, featuring just 1.51 M parameters and an AUC score of 0.981. This demonstrates the efficiency and effectiveness of MAM-Fusion-Net compared to larger models.

### *Deep classification using CFPs* + *VFs* + *OCT*

Since the CNN + S-I-C module works best for CFP and VF image data, this model was adopted for fusion with handcrafted OCT thickness features extracted using CIRRUS HD OCT. The features extracted by the CNN + S-I-C module from the dual combination of VF and CFP were further fused with the CIRRUS OCT-extracted features (CFP + VF + OCT), which produced the best performance among all the uni- (CFP/VF/OCT) and dual multimodal models (CFPs + VFs). The triple combination of CFP + VF + OCT results in a sensitivity of 0.970 (95% CI 0.938–1.000), specificity of 0.987 (95% CI 0.970–1.000), accuracy of 0.979 (95% CI 0.961–0.996), F1-score of 0.978 (95% CI 0.960–0.997), covering an area of 0.998 under the ROC curve (95% CI 0.996–1.000). In terms of number of parameters, inclusion of the OCT features did not increase the parameters significantly, keeping the count within 1.51 M.

### Sub-classification and comparative analysis

Once the optic neuropathy cases were classified by the top-level primary classifier, a sub-classification was performed on glaucoma vs non-glaucoma (neurodegenerative disease) diagnosis (Fig. 5, Supplementary Table 2). We trained and tested unimodal and multimodal classifiers for glaucoma vs non-glaucoma diagnosis for comparative analysis with fivefold cross-validation (Supplementary Table 2). For the unimodal approaches, the CFP-based CNN model generated a mean sensitivity of 0.793, specificity of 0.718, accuracy of 0.710, and F1-score of 0.789, covering an AUC of 0.785 across the folds. The VF-based CNN model produced comparatively superior performance to the CFP-based model, with a mean sensitivity of 0.805, specificity of 0.748, accuracy of 0.782, F1-score of 0.832, covering an AUC of 0.844 across the folds. Employing the multi-attention-based fusion using the S-I-C block, the MAM-Fusion-Net model outperformed the unimodal models with a mean sensitivity of 0.904 (95% CI 0.842–0.966), specificity of 0.910 (95% CI 0.854–0.966), accuracy of 0.897 (95% CI 0.846–0.948), F1-score of 0.910 (95% CI 0.864–0.956), covering an AUC of 0.960 (95% CI 0.931–0.989) across the folds.

A two-tailed paired t-test was performed to compare the performance of unimodal and multimodal models across fivefold cross-validation (Supplementary Table 5). For neuropathy diagnosis using the top-level classifier, a paired t-test between the CFP-based unimodal (CNN) and multimodal (CNN + S-I-C) MAM-Fusion-Net model shows a highly significant difference (p < 0.001) in terms of sensitivity (p = 0.00002), accuracy (p = 0.00003), AUC (p = 0.00017) and F1-score (p = 0.00001). Comparison of the VF-based unimodal model with the attention-based multimodal model shows a very significant (p < 0.01) difference in terms of sensitivity (p = 0.00127), accuracy (p = 0.00174), AUC (p = 0.00372) and F-1 score (p = 0.00178). A comparison of OCT based SVM model and OCT + CFP + VF using MAM-Fusion-Net shows a highly significant difference (p < 0.001) in terms of all metrics.

For glaucoma vs non-glaucoma using the sub-level classifier, a paired t-test (Supplementary Table 5) between the CFP-based unimodal (CNN) and multimodal (CNN + S-I-C) MAM-Fusion-Net model shows a highly significant (p < 0.001) difference in specificity (p = 0.00042) and a very significant difference (p < 0.01) in terms of accuracy (p = 0.00691), a significant difference in AUC (p = 0.01601), and a significant difference (p < 0.05) in F1 score (p = 0.00854). When comparing the VF-based unimodal model with the attention-based multimodal model, it shows a highly significant (p < 0.001) difference in specificity (p = 0.00091) and a very significant (p < 0.01) difference in terms of accuracy (p = 0.00811) and AUC (p = 0.00557), with a significant difference (p < 0.05) in F1 score (p = 0.01747).

## Discussion

The multiple unimodal and multimodal models implemented in this study revealed that the combination of spatial, self and cross-modal attention improves the performance of the diagnostic system for optic neuropathy and glaucoma diagnosis. The study results can be discussed concerning the performance of the OD and fovea localisation and unimodal and multimodal models’ implications of the findings, study limitations, and potential avenues for future research.

Several traditional image processing and deep learning-based methods have been developed in this study for OD and fovea localisation, and unimodal vs multimodal classifications. First, for OD localisation, the U-Net-based semantic segmentation model with an EfficientNetB1 backbone outperformed the sole EfficientNetB1-based regressor, achieving 11.6% higher IoU and approximately a sevenfold decrease in Euclidean distance between actual and predicted coordinates. This implies that rather than a coordinate prediction, it is comparatively efficient to segment the OD and then localise the centre of the disc. The performance of the semantic segmentation model for OD localisation (mean ED: 24 pixels in IDRID dataset) also outperforms the study by Babu et al.^64^ (mean ED: 80.48 using ResNet-18 and 60.32 pixels on IDRID dataset using ResNet-50) and Li et al.^65^ (mean ED: 32.60 using Region Proposal Network (RPN) with faster-RCNN and ResNet-50 as base). Second, for fovea localisation, the traditional approaches using Gaussian filtering and superpixeling produced an IoU ranging from 0.92 to 0.95, representing a 6.98% to 10.47% increase compared to the EfficientNetB1-based regressor (IoU of 0.86). Additionally, the traditional approaches resulted in Euclidean distances ranging from 2.48 to 3.45, corresponding to approximately a 2.53 to 1.82-fold decrease compared to the EfficientNetB1-based regressor (ED = 6.27) between actual and predicted coordinates. The performance of the traditional image processing-based approach (mean ED: 52.02 REFUGE2 dataset) outperformed the deep localisation models by the studies Babu et al.^64^ (mean ED: 95.45 pixels on IDRID dataset using ResNet-50) and 115.22 using ResNet-18)^64^, and comparable to Li et al.^65^ (mean ED: 52.00 using RPN with faster-RCNN and ResNet-50 as base in IDRID dataset). These results indicate the strength of the conventional image processing methods over deep learning methods in localisation tasks in medical imaging, which is far lower than the deep learning-based approaches.

Third, deep multimodal models combining CFPs and VFs significantly outperform unimodal models using CFPs (p < 0.001) and VFs (p < 0.01). Among these multimodal models, attention-based fusion models show superior performance compared to those without attention. Specifically, the combination of spatial, self, and cross-modal attention (S-I-C module) applied to a custom CNN network achieves better accuracy, AUC, and F1-score (p < 0.01) than the CNN model without attention, yielding an optimised and efficient model with an AUC of 0.98 (95% CI 0.96–1.00) for neuropathy diagnosis and 0.96 (95% CI 0.93–0.99) for glaucoma subclassification, while using only 1.51 M trainable parameters. The S-I-C module introduced in this study is highly effective in capturing spatial hierarchies, long-range dependencies, and intermodal correlations, ensuring optimal contribution from both modalities to the final fused representation. Combining OCT with CFP and VF modalities using the MAM-Fusion-Net boosts the performance of neuropathy diagnosis, with a sensitivity of 0.970 (95% CI 0.938–1.000), specificity of 0.987 (95% CI 0.970–1.000), accuracy of 0.979 (95% CI 0.961–0.996), F1-score of 0.978 (95% CI 0.960–0.997), and covers an area of 0.998 under the ROC curve (95% CI 0.996–1.000). In terms of number of parameters, including the OCT features did not increase the number of parameters significantly, keeping the count within 1.51 M, which means that the model’s complexity and computational requirements remained manageable despite incorporating additional OCT data.

Lastly, the multi-attention-based multimodal approach demonstrated superior performance compared to other uni- and multi-modal methods for diagnosing glaucoma and neurodegenerative diseases. Among unimodal studies, OCT remains the most commonly used modality for evaluating structural damage caused by glaucoma, enabling the selection of a limited set of RNFL, GC-IPL, and MC thickness features for traditional machine learning classifiers with explainability techniques. For instance, Wu et al.^36^ employed an SVM classifier with 114 OCT features (Spectralis OCT) and achieved an AUC of 0.82 for glaucoma diagnosis. Similarly, Kooner et al.^66^ utilised 64 OCTA and other clinical features with an XGBoost classifier, attaining an overall accuracy of 83.9% in distinguishing glaucoma from normal cases. Xu et al.^37^ leveraged 3D circumpapillary RNFL (cpRNFL) thickness measurements (four quadrants) alongside 68 super-pixel-based features, achieving AUCs ranging from 0.71 to 0.86 in differentiating normal participants from glaucoma suspects.

For neurodegenerative disease studies, Wang et al.^67^ used OCT features to distinguish dementia patients (159 cases) from normal individuals (299 cases), obtaining test AUCs between 0.52 and 0.75 across seven supervised ML classifiers. Diaz et al.^68^ achieved AUCs of 0.69 and 0.71 in two separate datasets when differentiating Parkinson’s patients from normal controls using an SVM classifier. In contrast, our approach, utilising 45 spatial domain features, achieved an AUC of 0.892 (95% CI 0.855–0.921) for distinguishing neuropathy from normal cases and an AUC of 0.820 (95% CI 0.788–0.853) for classifying glaucoma versus non-glaucoma cases using a SVM classifier.

Moreover, our multimodal model outperformed the multimodal-based classification models by Shin et al.^69^ using ultra-wide-field (UWF) fundus photographs (AUC: 0.90, VGG-19 network) and true-colour confocal scan (TCCS) (AUC: 0.87, VGG-19 network) separately for glaucoma diagnosis (glaucoma vs normal), Oh et al.^70^ (AUC: 0.930, XGBoost) using combined information from VF test, Spectralis OCT and intraocular pressure (IOP) test for glaucoma diagnosis (glaucoma vs normal), Oh et al.^71^ (AUC range 0.890–0.950, RF, SVM and XGBoost) using the fusion of OCT, VF, CFPs and clinical information for glaucoma vs normal classification, and Wisely et al.^72^ (AUC: 0.840, custom CNN) using the multimodalities OCT, OCTA, UWF and Fundus Autofluorescence (FAF) for dementia diagnosis. Conversely, the OCT, VF and CFP-based multimodal model achieved an area under the sensitivity–specificity curve (AUC) of 0.998 (95% CI 0.996–1.000) for diagnosing neuropathy versus normal participants and 0.981 (95% CI 0.966–0.996) for glaucoma versus non-glaucoma. However, this study incorporates a unique patient cohort, particularly including cases of ION, dementia, and Parkinson’s disease within the non-glaucoma group under neuropathy. As a result, the model’s performance cannot be directly compared with existing studies on neuropathy diagnosis. Additionally, we acknowledge that direct comparisons with prior machine and deep learning studies are constrained by variations in datasets, input types, and experimental setups. A benchmarking study using the same dataset and evaluation protocol would be necessary for a more precise assessment of relative performance.

This study offers significant insights into multimodal data preprocessing and intermediate fusion for medical diagnosis, more specifically in glaucoma and neurodegenerative disease diagnosis. The research implications from this study could be discussed in terms of multimodal data preprocessing, intermediate fusion and glaucoma diagnosis. First, applying transfer learning to a private dataset proved effective for OD and fovea localisation, streamlining necessary preprocessing for multimodal fusion and reducing the need for costly and time-consuming data annotations^48^. Second, traditional image processing methods demonstrated greater robustness than deep learning approaches for fovea localisation, lowering computational costs by reducing the reliance on GPU or TPU resources for training deep learning models. Third, incorporating multi-attention mechanisms in multimodal models significantly enhances performance, with dual-attention (spatial-self attention module) outperforming single-attention (spatial attention module), and triple-attention mechanisms (spatial-self-cross-modal attention module, S-I-C) further boosting performance by capturing spatial hierarchies, long-range dependencies, and intermodal correlations. These modules can be adapted for other multimodal applications in medical and non-medical domains based on the dataset characteristics. Fourth, the proposed S-I-C attention module can be plugged into fine-tuned shallow CNN architectures such as VGG-Net and LeNet, or lightweight networks like MobileNet, depending on data size and nature. This approach can leverage transfer learning strategies and pre-trained weights but also capture the long-term dependency using our proposed multi-attention module. Finally, the developed multi-attention-based multimodal networks showed promise in neuropathy diagnosis and also differentiation of glaucoma and non-glaucomatous optic neuropathies, including neurodegenerative diseases, outperforming the state-of-the-art methods in this area^3^. The developed model can be deployed in a clinician’s computer or imaging devices used to make clinical decisions based on combined multimodalities.

This study stands out for its use of a unique patient cohort, including both glaucoma, ischaemia-induced and neurodegenerative diseases, and a novel approach to multimodal data preprocessing and fusion. The inclusion of disc-fovea angle identification and alignment for combining CFP and VF data addresses a critical gap in existing research, where such preprocessing steps are often ignored^28^. This study also introduces an efficient method for fovea localisation using traditional image processing, outperforming deep learning methods. In terms of multimodal fusion, most existing approaches rely on early or late fusion, which limits modality-specific feature extraction and cross-modal interactions^30–32^. To address this, we developed an intermediate fusion strategy incorporating spatial, self, and cross-modal attention mechanisms. These attention mechanisms enable the model to focus on relevant features and interactions across modalities, enhancing interpretability, robustness, and classification performance. Our proposed multi-attention-based multimodal fusion network (MAM-Fusion-Net) outperforms single or dual-attention mechanisms, marking a notable contribution to the field. By integrating these attention mechanisms, the model effectively utilises structural and functional data sources, significantly advancing the performance of multimodal fusion in medical diagnosis.

While our proposed stacked multi-attention mechanism offers clear advantages, several limitations should be noted. First, the dataset used was private, lacking annotated data for OD and fovea localisation, as well as angle calculation. To address this, a transfer learning approach was employed, training the localisation models on public datasets (i.e. IDRID, ORIGA and REFUGE datasets) and testing on private CFEH images. Second, the study was constrained to patients with VF, CFP and OCT data, which reduced the sample size. Cases where only one modality was available were excluded. Although mean image imputation could address missing modalities, it might compromise model performance. A more robust solution would involve using generative models, such as generative adversarial networks (GANs) or diffusion models, to generate the missing modality for multimodal fusion^73^.

Third, integrating multi-attention mechanisms significantly increases computational complexity, leading to a higher number of trainable parameters, particularly when using state-of-the-art architectures. We evaluated our multi-attention module on more complex architectures like ResNet-50 and DenseNet-121. However, these models exhibited overfitting, likely due to their intricate designs, including residual connections and batch normalisation, which may have limited their ability to effectively capture long-range dependencies in our relatively small multimodal dataset. In light of this, our proposed stacked spatial, self, and cross-modal attention module is more suited to fine-tuned, shallower CNN architectures, such as VGG-Net and LeNet, or lightweight models like MobileNet. To further reduce model complexity, employing knowledge distillation techniques or advanced optimization algorithms could help develop a more efficient, lightweight model^74^. Future research could also incorporate additional clinical data (e.g. age, gender, diagnosis history) and textual information such as optometrist notes, providing more interpretable results for clinicians. Fourth, while the increased complexity of the proposed MAM-Fusion-Net architecture compared to simpler and explainable models using traditional machine learning with a single modality (especially using OCT^35^) contributes to improved performance, it also poses challenges for implementation and deployment, particularly for researchers or clinicians with limited computational resources. Future work could explore ways to balance model performance and complexity by leveraging more efficient architectures without compromising predictive accuracy.

Fifth, the multi-attention multimodal model using the S-I-C module in this study may be limited by the specific devices (TopCon for fundus images) and methods (24–2 VFs) used to collect the neuropathy data. Expanding the model to include data from other imaging devices and VF methods could improve generalisability. Additionally, testing the S-I-C module on other multimodal medical data or different domains would further validate its applicability. Finally, due to the unavailability of public multimodal data (OCT, VF and CFPs) for the given cohort, we could not validate our model on an independent dataset. The limited sample size constrains a thorough evaluation of the model’s ability to generalise, even though five-fold cross-validation was applied. Validation using a larger, independent external clinical dataset is necessary to further establish the model’s robustness and reliability.

## Supplementary Information

Supplementary Information.

## Data Availability

The raw private data are not available for public access, but the statistically analysed VF data in tabular format for a group of patient cohorts (normal, glaucoma, non-glaucoma including the neurodegenerative diseases) may be accessible upon reasonable request to the corresponding author. Used public dataset in this study: IDRID Dataset; DOI: 10.3390/data3030025; Date accessed: 30 May 2024; license: CC BY 4.0. ORIGA dataset; DOI: 10.1109/IEMBS.2010.5626137; Date accessed: 30 May 2024; license: CC BY 4.0. REFUGE DatasetL 10.1016/j.media.2019.101570; Date accessed: 30 May 2024; license: CC BY 4.0.

## Code availability

The code of this work can be found in 10.5281/zenodo.18906936.
