# Supplementary material for: Multiple attention based deep multimodal fusion network for glaucoma and neurodegenerative disease diagnosis
Source: Sci Rep. 2026 Apr 26;16:19247. doi: 10.1038/s41598-026-46855-6 (PMC13284329; doi:10.1038/s41598-026-46855-6)
Supplement: Supplementary file 1 — Supplementary Information. [file 41598_2026_46855_MOESM1_ESM.pdf]

Supplementary Information

Multiple attention based deep multimodal fusion network for glaucoma and neurodegenerative disease diagnosis

Md Mahmudul Hasan[a,*], Jack Phu[b,c,d,e], Henrietta Wang[b,c,e], Michael Kalloniatis[b,e,f], Arcot Sowmya[a], Erik Meijering[a]

[a] School of Computer Science and Engineering, University of New South Wales, Sydney, NSW, Australia
[b] School of Optometry and Vision Science, University of New South Wales, Sydney, NSW, Australia
[c] Centre for Eye Health, University of New South Wales, Sydney, NSW, Australia
[d] Faculty of Medicine and Health, University of Sydney, Camperdown, NSW, Australia
[e] School of Medicine (Optometry), Deakin University, Waurn Ponds, VIC, Australia
[f] University of Houston College of Optometry, University of Houston, Houston, TX, United States of America

[*] *Corresponding author*
Md Mahmudul Hasan
School of Computer Science and Engineering
University of New South Wales, Sydney, NSW 2052, Australia
Email: md_mahmudul.hasan@unsw.edu.au

Short title: Multiple attention based deep multimodal fusion network

Keywords: 24-2 test grid, colour fundus photographs, glaucoma, dementia, Parkinson’s disease, perimetry, visual fields

Supplementary Algorithm

Input: I(x, y): RGB retinal fundus image
Output: f_x: X-coordinate of the foveal centre (integer), f_y: Y-coordinate of the foveal centre (integer)
Parameters: T: Threshold value for intensity-based segmentation (integer), N: Number of superpixels for SLIC segmentation (integer), σ: Standard deviation for Gaussian filter (float)

Steps:

1. Convert the image to grayscale:
   $I_{gray}(x, y)$ = Convert COLOR_GBR2Gray ($I(x, y)$)).
2. Define the radius r of the circular region centred at (c_x, c_y)
   r = r/3
3. Extract a sub-image corresponding to the circular region:
   $I_{circ}(x, y) = I_{gray}(c_y - r\colon\ c_y + r, c_x - r\colon\ c_x + r\ )$
4. Apply SLIC superpixel segmentation to $I_{circ}(x, y)$ with N segments:
   S (x, y) = slic(I_{circ}(x, y), n_segments=N).
5. Identify the darkest superpixel:
   Initialise min_I and label_darkest

   For each unique superpixel label l in N:
   - Create a mask $M_l(x, y) = (S\ (x, y) == l)$.
   - Calculate mean intensity within the mask:
     $$\frac{\sum(I_{circ}(x, y)\ .\ M_l(x, y))}{\sum\ M_l(x, y)}$$
   - If I_{mean_l} < min_I and I_{mean_l} <= T (exclude high intensity):
     - Update min_I = I_{mean_l} and label_darkest = l

   End For
6. If label_darkest is found:
   - $M_{darkest}$(x, y) = S (x, y) == label_darkest) [Create mask (for the darkest superpixel)]
   - (d_x, d_y) = index (argmin (I_{circ}(x, y) * $M_{darkest}$(x, y)), $M_{darkest}$.shape). [Find the darkest pixel]
   - I_{filt}(x, y) = gaussian_filter (I_{circ}(x, y), σ). [a Gaussian filter to I_{circ}(x, y) centred at (d_x, d_y) with standard deviation σ]
   - Find local minima coordinates:
     minima_coords $I_{\_filt}$(x, y) == MIN($I_{\_filt}$).
     f_x = MEAN (minima_coords[:, 0]) + c_x – r [mean of local minima coordinates and adjust offset from original centre]
     f_y = MEAN (minima_coords[:, 1]) + c_y – r

   else go to step 5.

Output: The algorithm returns the coordinates (f_x, f_y) representing the estimated location of the fovea.
Explanation:
Symbols are used to represent image data (I), grayscale image (I_{gray}), circular region (I_{circ}), superpixel segmentation (S), masks (M_l, $M_{\_darkest}$), filtered image (I_{filt}), and coordinates (c_x, c_y, d_x, d_y, f_x, f_y).

Algorithm-1: Fovea localisation using the combined approach of superpixels and gaussian filtering.

Supplementary Figures

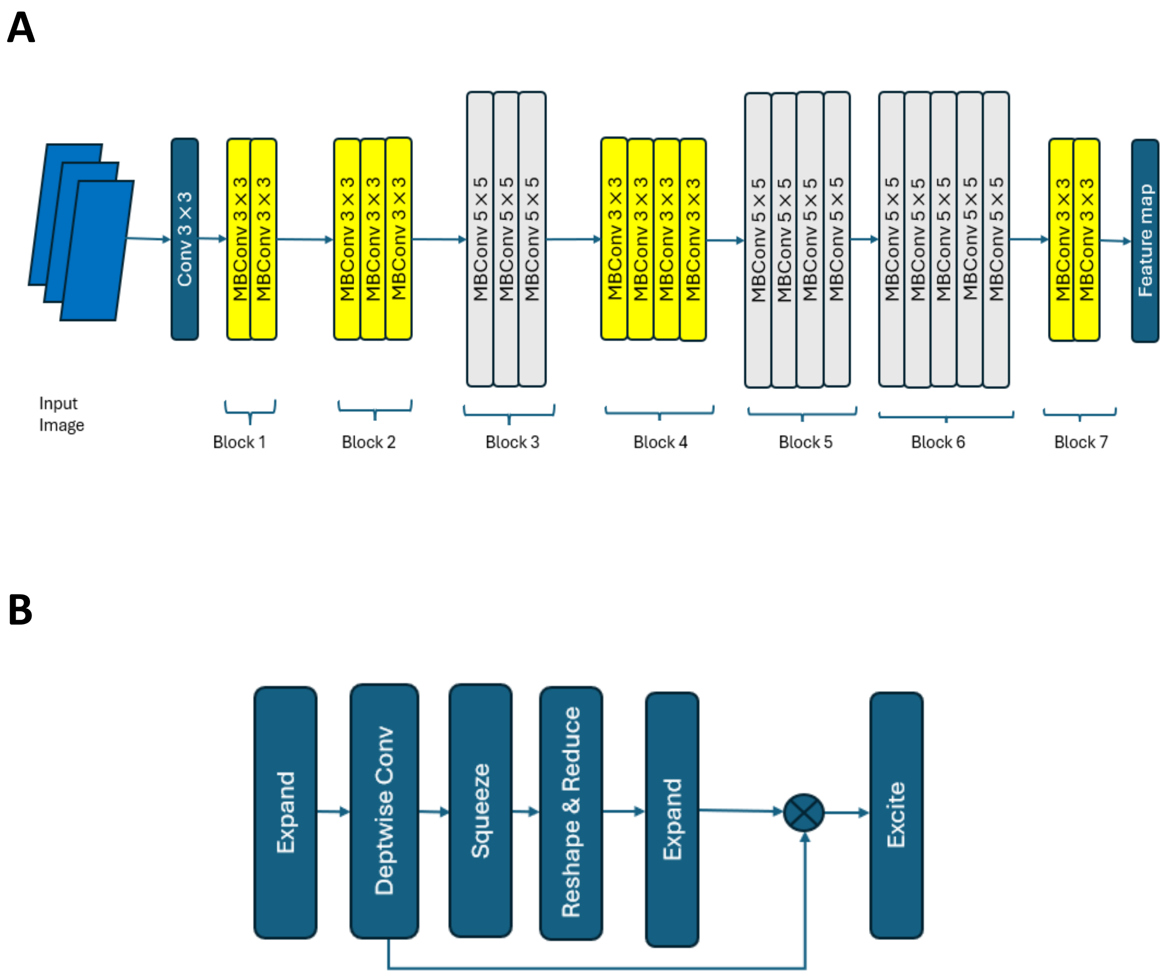

Supplementary Figure 1. The EfficientNetB1 architecture for optic disc localisation, with MBConv as its fundamental building blocks. (A) The entire EfficientNetB1 architecture consists of seven distinct blocks. (B) The primary component of the network is the MBConv (mobile inverted bottleneck convolution) [1].

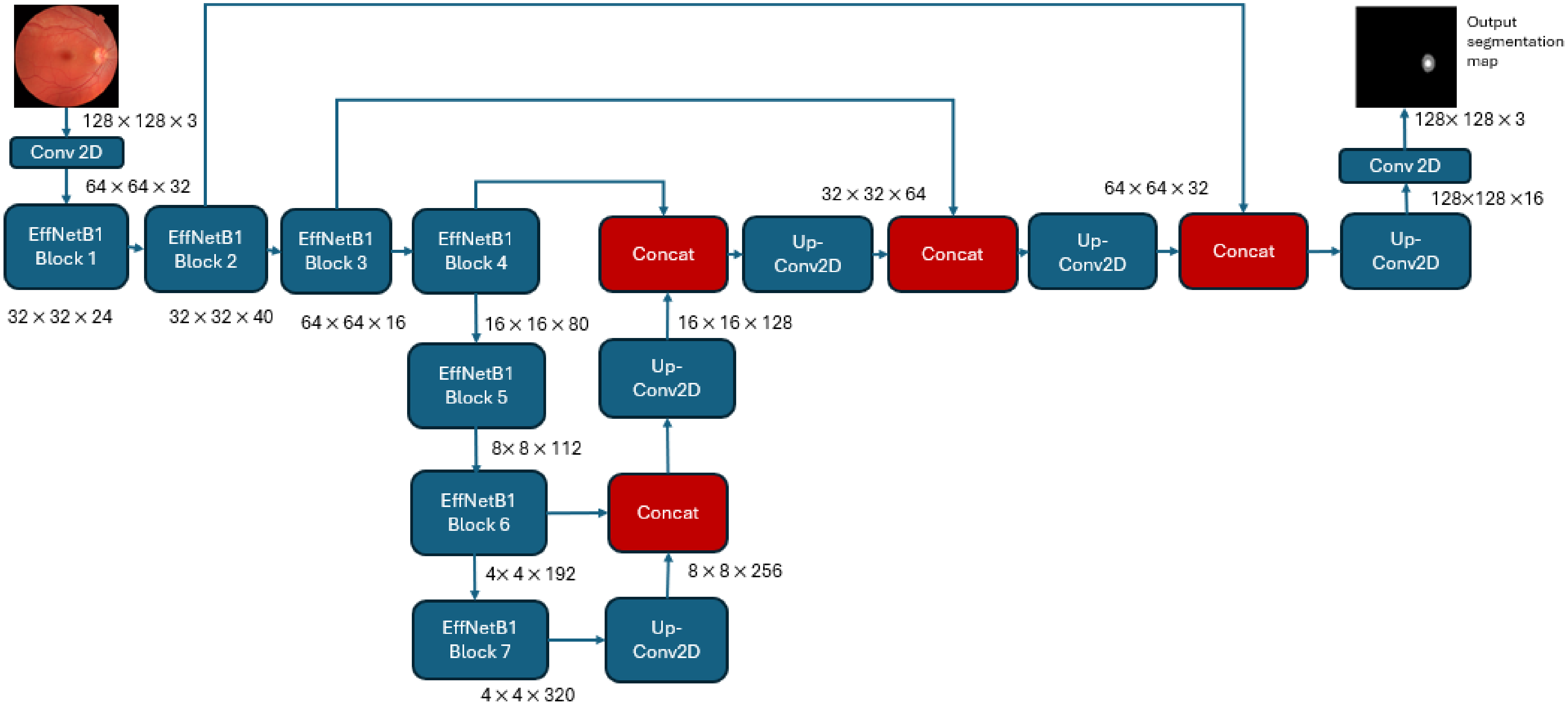

Supplementary Figure 2. EfficientNetB1-UNet for semantic segmentation of optic disc and cup, which was finally used to predict optic disc coordinate.

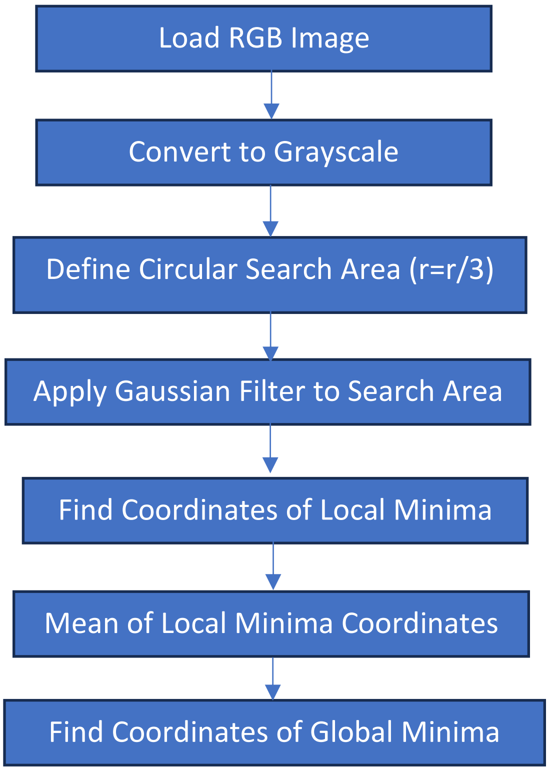

Supplementary Figure 3. Flow chart illustrating fovea localisation using Gaussian filtering.

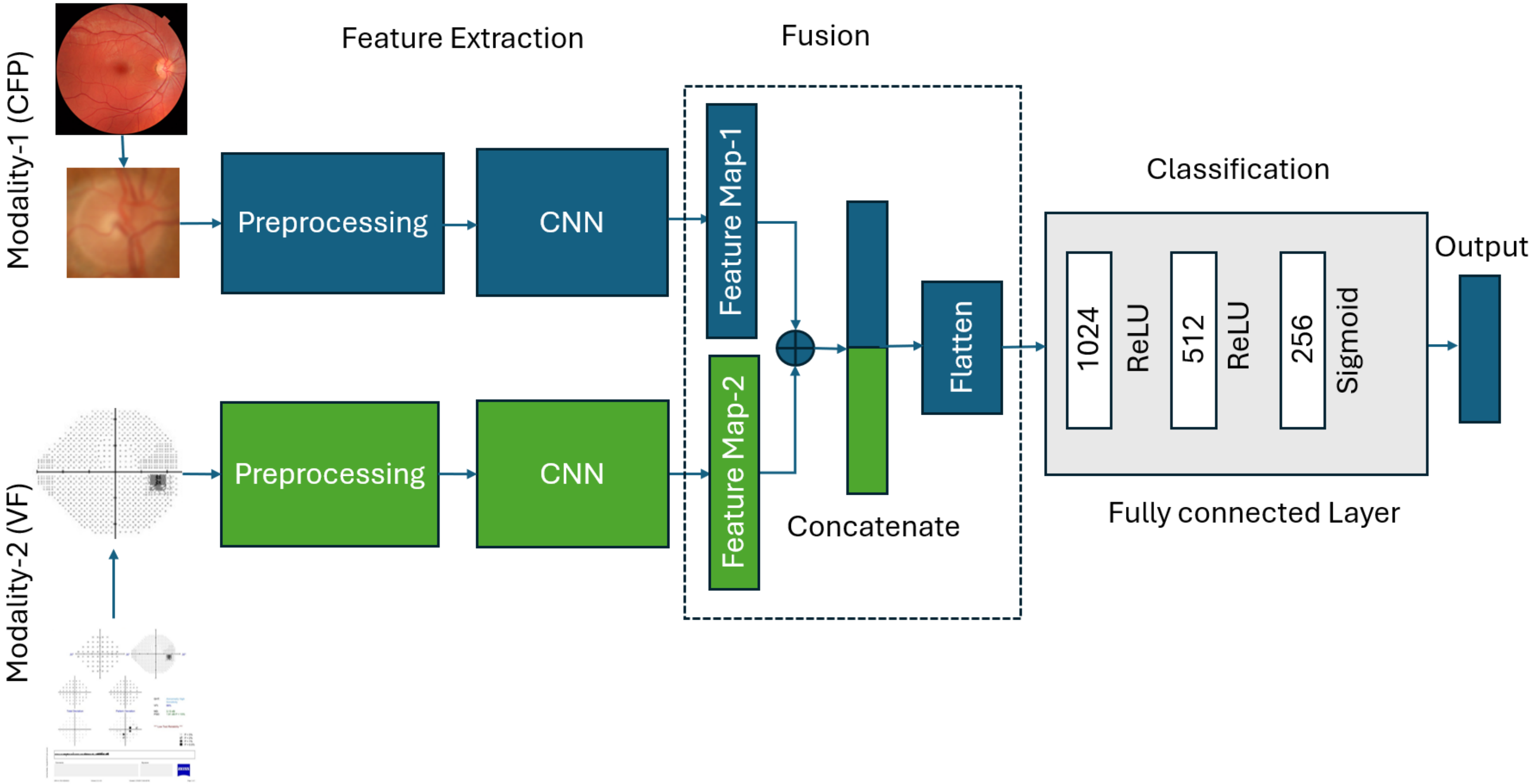

Supplementary Figure 4. Schematic of the base multimodal model. Feature map-1 and feature map-2 are extracted using CNN networks, followed by a concatenation for feature fusion. The number of neurons in the fully connected classification layer was tuned.

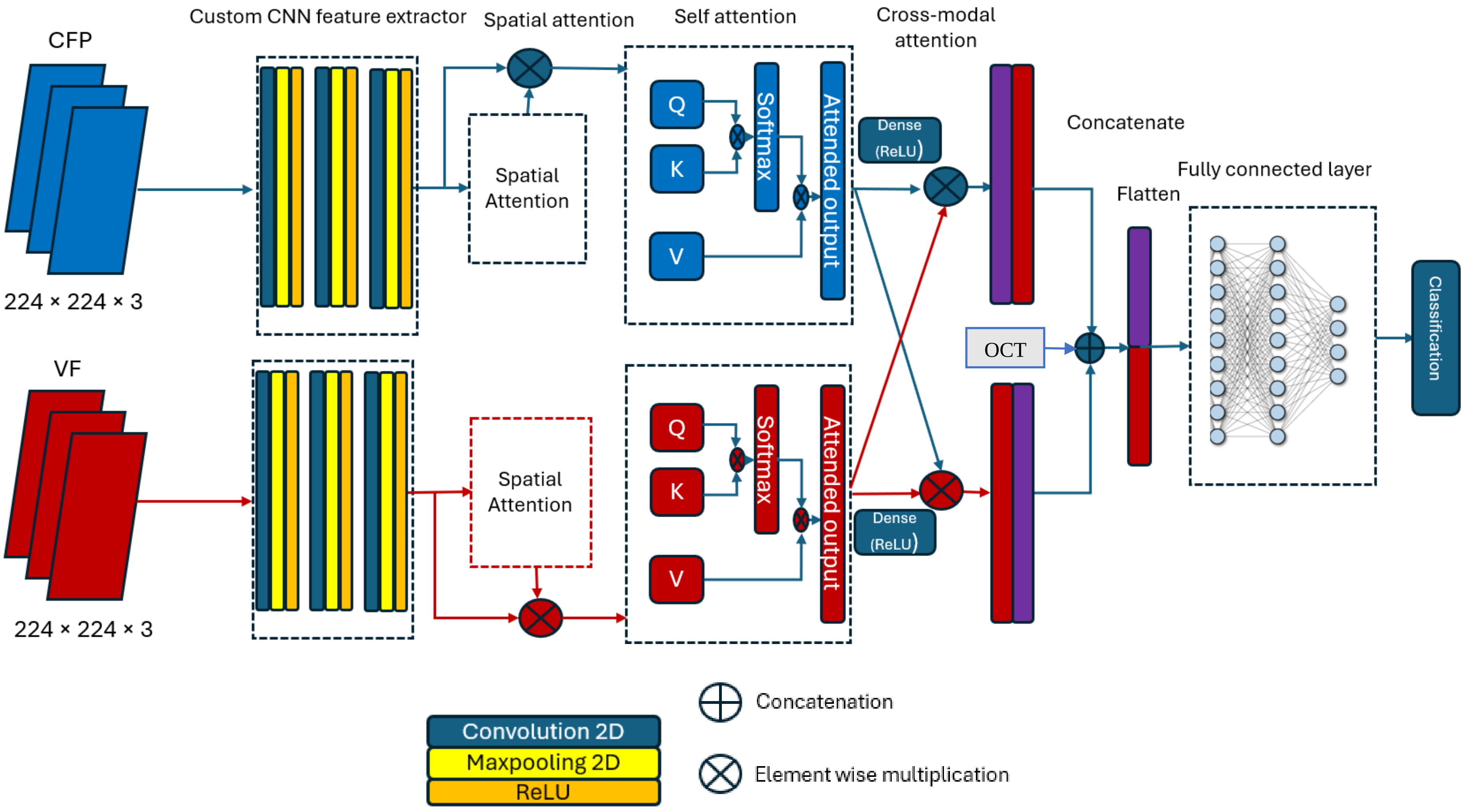

Supplementary Figure 5. Multi-attention-based deep multimodal fusion network (MAM-Fusion-Net) for neuropathy diagnosis using OCT, colour fundus photographs and visual fields. The CNN-extracted features are passed through the Spatial + Self + Cross-modal attention module (S-I-C module). The OCT features are concatenated at the final stage before classification. CFP: Colour Fundus Photograph, VF: Visual Fields.

Supplementary Tables

RNFL Thickness Analysis	GC-IPL Thickness Analysis	Macular Thickness Analysis
RNFL symmetry (%)	Average GC-IPL thickness	ILM-RPE thickness-central subfield
Average RNFL thickness (µm)	Minimum GC-IPL thickness (µm)	ILM-RPE thickness-volumetric cube
RNFL thickness ring area (mean) (µm)	GC-IPL superior (µm)	ILM-RPE thickness-average cube
RNFL thickness in quadrants (µm) (count=4)	GC-IPL superonasal (µm)	ILM-RPE centre-foveal
• RNFL superior	GC-IPL inferonasal (µm)	ILM-RPE inner superior (iSup)
• RNFL nasal	GC-IPL inferior (µm)	ILM-RPE inner nasal (iNas)
• RNFL inferior	GC-IPL inferotemporal (µm)	ILM-RPE inner inferior (iInf)
• RNFL tempora	GC-IPL-superotemporal (µm)	ILM-RPE inner temporal (iTem)
RNFL clock hours		ILM-RPE outer superior (oSup)
RNFL clock hour 1- 12 (count= 12)		ILM-RPE outer nasal (oNas)
Rim area		ILM-RPE outer inferior (oInf)
Disc area		ILM-RPE outer temporal (oTem)
Average cup to disc ratio		
Vertical cup to disc ratio		
Cup volume		
Neuro-retinal Rim Thickness		
Count (individual analysis) =25	8	12
Total Count= 45		

Supplementary Table 1: Spatial domain features extracted using CIRRUS HD OCT software for VF prediction from OCT.

		Neuropathy vs Normal subjects					Glaucoma vs Non-Glaucoma				
**Modality (Method)**	**Fold**	**Sensitivity**	**Specificity**	**Accuracy**	**AUC**	**F1-score**	**Sensitivity**	**Specificity**	**Accuracy**	**AUC**	**F1-score**
CFP (CNN)	1	0.627	0.750	0.684	0.751	0.681	0.810	0.692	0.528	0.553	0.691
	2	0.756	0.800	0.779	0.834	0.764	0.804	0.692	0.667	0.726	0.755
	3	0.654	0.651	0.653	0.751	0.673	0.835	0.808	0.847	0.907	0.887
	4	0.652	0.792	0.723	0.798	0.698	0.825	0.677	0.746	0.876	0.786
	5	0.647	0.907	0.766	0.898	0.750	0.691	0.720	0.761	0.863	0.828
	Mean ± SD	0.667 ± 0.05	0.780 ± 0.092	0.721 ± 0.053	0.807 ± 0.062	0.713 ± 0.041	0.793 ± 0.058	0.718 ± 0.052	0.710 ± 0.120	0.785 ± 0.147	0.789 ± 0.074
	95% CI	0.623 – 0.711	0.699 – 0.861	0.675 – 0.767	0.753 – 0.861	0.677 – 0.749	0.742 – 0.844	0.672 – 0.764	0.605 – 0.815	0.656 – 0.914	0.724 – 0.854
VF (CNN)	1	0.725	0.750	0.737	0.811	0.747	0.834	0.747	0.764	0.796	0.813
	2	0.711	0.800	0.811	0.829	0.780	0.791	0.677	0.778	0.859	0.837
	3	0.481	0.830	0.684	0.712	0.625	0.783	0.738	0.694	0.747	0.766
	4	0.696	0.838	0.819	0.865	0.790	0.725	0.777	0.817	0.888	0.851
	5	0.784	1.000	0.883	0.959	0.879	0.891	0.800	0.859	0.928	0.891
	Mean ± SD	0.679 ± 0.116	0.844 ± 0.094	0.787 ± 0.077	0.835 ± 0.089	0.764 ± 0.092	**0.805 ± 0.062**	0.748 ± 0.047	**0.782 ± 0.062**	**0.844 ± 0.072**	**0.832 ± 0.046**
	95% CI	0.577 – 0.781	0.762 – 0.926	0.720 – 0.854	0.757 – 0.913	0.683 – 0.845	0.751 – 0.859	0.707 – 0.789	0.728 – 0.836	0.781 – 0.907	0.792 – 0.872
OCT (SVM)	1	0.729	0.848	0.787	0.859	0.778	0.571	0.793	0.662	0.761	0.667
	2	0.875	0.739	0.809	0.893	0.824	0.837	0.821	0.831	0.829	0.857
	3	0.792	0.87	0.83	0.888	0.826	0.628	0.929	0.746	0.850	0.750
	4	0.653	0.867	0.755	0.865	0.736	0.721	0.821	0.761	0.812	0.785
	5	0.878	0.911	0.894	0.94	0.896	0.595	0.893	0.714	0.849	0.714
	Mean ± SD	**0.785 ± 0.096**	**0.847 ± 0.065**	**0.815 ± 0.052**	**0.889 ± 0.032**	**0.812 ± 0.06**	0.671 ± 0.109	**0.851 ± 0.057**	0.743 ± 0.062	0.820 ± 0.037	0.755 ± 0.072
	95% CI	0.701 – 0.870	0.790 – 0.903	0.769 – 0.860	0.861 – 0.917	0.759 – 0.864	0.575 – 0.766	0.802 – 0.901	0.688 – 0.797	0.788 – 0.853	0.691 – 0.818
CFP + VF [**MAM-Fusion-Net**]	1	0.843	0.932	0.884	0.948	0.887	0.816	0.971	0.889	0.926	0.886
	2	0.956	0.980	0.968	0.974	0.966	0.891	0.846	0.875	0.955	0.901
	3	0.942	0.930	0.937	0.988	0.942	0.861	0.923	0.819	0.931	0.843
	4	0.935	0.979	0.957	0.994	0.956	0.975	0.968	0.972	0.998	0.975
	5	0.922	1.000	0.957	1.000	0.959	0.978	0.840	0.930	0.990	0.947
	Mean ± SD	0.919 ± 0.044	0.964 ± 0.031	0.941 ± 0.034	0.981 ± 0.02	0.942 ± 0.032	0.904 ± 0.071	0.910 ± 0.064	0.897 ± 0.058	0.960 ± 0.033	0.910 ± 0.052
	95% CI	0.880 – 0.958	0.937 – 0.991	0.911 – 0.971	0.963 – 0.999	0.914 – 0.970	0.842 – 0.966	0.854 – 0.966	0.846 – 0.948	0.931 – 0.989	0.864 – 0.956
OCT + CFP + VF [MAM-Fusion-Net]	1	0.957	0.957	0.957	0.997	0.957	0.902	0.900	0.901	0.964	0.914
	2	1.000	0.977	0.989	1.000	0.990	0.952	0.897	0.930	0.988	0.941
	3	1.000	1.000	1.000	1.000	1.000	0.867	0.962	0.901	0.961	0.918
	4	0.913	1.000	0.957	0.995	0.955	0.975	1.000	0.986	0.994	0.987
	5	0.980	1.000	0.989	0.999	0.990	0.978	1.000	0.986	0.999	0.989
	Mean ± SD	**0.970 ± 0.036**	**0.987 ± 0.019**	**0.979 ± 0.02**	**0.998 ± 0.002**	**0.978 ± 0.021**	**0.935 ± 0.049**	**0.952 ± 0.051**	**0.941 ± 0.043**	**0.981 ± 0.017**	**0.95 ± 0.037**
	95% CI	0.938 – 1.002	0.970 – 1.004	0.961 – 0.996	0.996 – 1.000	0.960 – 0.997	0.892 – 0.977	0.907 – 0.996	0.903 – 0.978	0.966 – 0.996	0.918 – 0.982

Supplementary Table 2: Comparative of unimodal and multimodal models for optic neuropathy (glaucoma, ischaemic optic neuropathy, dementia and Parkinson's disease) diagnosis using the top-level classifier and glaucoma and non-glaucoma (ischaemic optic neuropathy, dementia and Parkinson's disease) diagnosis using the sub-level classifiers.

Type	Attention mechanism	Model	Total parameters (M)	Trainable Parameters (M)	Non-trainable Parameters (M)
Unimodal (VF/CFP)	No Attention	CNN	**0.03**	**0.03**	**0**
		VGG	0.46	0.20	0.26
		MobileNet-v1	0.24	0.19	0.04
		LeNet-5	0.08	0.08	0
		ViT-16	85.82	85.82	0
Multimodal (CFP + VF)	No Attention (concatenation)	CNN	0.06	0.06	0
		VGG-16	0.90	0.90	0
		MobileNet-v1	**0.06**	**0.06**	0
		LeNet-5	0.90	0.90	0
		Siamese	0.06	0.06	0
	Vision Transformer (multi-head self attention)	ViT-16	85.82 (shared)	85.82 (shared)	0
	Single Attention	CNN + Spatial attention	2.99	2.99	0
	Dual attention	CNN + Spatial + Self attention	**1.51**	**1.51**	**0**
	Triple attention	CNN + Spatial + Self + Cross-modal attention	**1.51**	**1.51**	**0**
		VGG16 + Spatial + Self + Cross-modal attention	30.04	10.04	19.99
		MobileNet + Spatial + Self + Cross-modal attention	7.67	7.62	0.04
		LENet + Spatial + Self + Cross-modal attention	5.27	5.27	0
		Siamese + Spatial + Self + Cross-modal attention	16.64	16.64	0
Multimodal (OCT+ CFP + VF)	Triple attention	CNN + Spatial + Self + Cross-modal attention	**1.51**	**1.51**	**0**

Supplementary Table 3: Number of total, trainable and non-trainable parameters in the developed models. Bolded numbers represent the lowest number of parameters within that type.

Comparison modals	p-value (two-tailed paired t-test)					Significance level				
	Sensitivity	Specificity	Accuracy	AUC	F1-score	Sensitivity	Specificity	Accuracy	AUC	F1-score
CNN vs CNN + Spatial	0.249	0.115	0.304	0.194	0.406	ns	ns	ns	ns	ns
CNN vs CNN + Spatial + Self	0.08	0.155	0.016	0.033	0.017	ns	ns	*	*	*
CNN vs CNN + Spatial + Self + Cross-modal attention [**MAM-Fusion-Net**]	**0.016**	**0.045**	**0.00098**	**0.004**	**0.001**	*****	*****	*******	******	******
VGG-16 vs VGG-16 + Spatial + Self + Cross-modal attention	0.129	0.015	0.027	0.021	0.030	ns	*	*	*	*
MobileNet-v1 vs MobileNet-v1 + Spatial + Self + Cross-modal attention	0.113	0.071	0.009	0.009	0.004	ns	ns	**	**	**
LeNet-5 vs LeNet-5 + Spatial + Self + Cross-modal attention	0.293	0.027	0.059	0.226	0.113	ns	*	ns	ns	ns
Siamese vs Siamese + Spatial + Self + Cross-modal attention	0.122	0.122	0.002	0.051	0.031	ns	ns	**	ns	*
ViT-16 vs ViT-16 + Spatial + Self + Cross-modal attention	0.226	0.276	0.153	0.373	0.135	ns	ns	ns	ns	ns

Supplementary Table 4: Results for two-tailed paired t-test for intra-multimodal models (across 5-folds) for neuropathy (glaucoma, ischaemic optic neuropathy, dementia and Parkinson’s disease) diagnosis. Note: $p<0.001$,"highly significant" (***), $p<0.01$,"very significant" (**), $p<0.05$,"significant" (*), $p>=0.05$, "not significant" (ns); S: Spatial module; S-I: Spatial + Self-attention module; S-I-C: spatial + self + cross-modal attention module.

		p-value (paired t-test)					Significance level				
	**Comparison**	Sensitivity	Specificity	Accuracy	AUC	F1-score	Sensitivity	Specificity	Accuracy	AUC	F1-score
Neuropathy vs Normals	CFP (CNN) vs CFP + VF (CNN + Spatial + Self + Cross)	**0.00002**	**0.00146**	**0.00003**	**0.00017**	**0.00001**	***	**	***	***	***
	VF (CNN) vs CFP + VF (CNN + Spatial + Self + Cross)	0.00127	0.01305	0.00174	0.00372	0.00178	**	*	**	**	**
	OCT (SVM) vs OCT + (CFP + VF) (CNN + Spatial + Self + Cross)	0.00195	0.00096	0.00014	0.00081	0.0002	**	***	***	***	***
Glaucoma vs Non-glaucoma	CFP (CNN) vs CFP + VF (CNN + Spatial + Self + Cross)	**0.01348**	**0.00042**	**0.00691**	**0.01601**	**0.00854**	*	***	**	*	**
	VF (CNN) vs CFP + VF (CNN + Spatial + Self + Cross)	0.02315	0.00091	0.00811	0.00557	0.01747	*	***	**	**	*
	OCT (SVM) vs OCT + (CFP + VF) (CNN + Spatial + Self + Cross)	0.0002	0.00049	0.00002	0.0000025	0.00008	***	***	***	***	***

Supplementary Table 5: Results of two-tailed paired t-test (across 5-folds) between unimodal and multimodal models for primary (neuropathy vs normal subjects) and secondary (glaucoma vs non-glaucoma) classifiers. Note: p<0.001,"highly significant" (***), p<0.01,"very significant" (**), p<0.05,"significant" (*), p>=0.05, "not significant" (ns). CFP: Colour Fundus Photographs, VF: Visual Fields, OCT: Optical Coherence Tomography. Neuropathy cohort includes patients with glaucoma, ischaemic optic neuropathy, dementia and Parkinson’s disease. Non-glaucoma cohort includes ischaemic optic neuropathy, dementia and Parkinson’s disease.

Reference

1 Baheti, B., Innani, S., Gajre, S. & Talbar, S. in *Proceedings of the IEEE/CVF Conference on Computer Vision and Pattern Recognition Workshops.* 358-359.
